# Modelling the impact and cost‐effectiveness of non‐governmental organizations on HIV and HCV transmission among people who inject drugs in Ukraine

**DOI:** 10.1002/jia2.26073

**Published:** 2023-04-03

**Authors:** Jack Stone, Adam Trickey, Josephine G. Walker, Sandra Bivegete, Nadiya Semchuk, Yana Sazonova, Olga Varetska, Frederick L. Altice, Tetiana Saliuk, Peter Vickerman

**Affiliations:** ^1^ Population Health Sciences University of Bristol Bristol UK; ^2^ Alliance for Public Health Kyiv Ukraine; ^3^ Yale University School of Medicine New Haven Connecticut USA

## Abstract

**Introduction:**

People who inject drugs (PWID) in Ukraine have high prevalences of HIV and hepatitis C virus (HCV). Non‐governmental organizations (NGOs) provide PWID with needles/syringes, condoms, HIV/HCV testing and linkage to opioid agonist treatment (OAT) and antiretroviral therapy (ART). We estimated their impact and cost‐effectiveness among PWID.

**Methods:**

A dynamic HIV and HCV transmission model among PWID was calibrated using data from four national PWID surveys (2011–2017). The model assumed 37–49% coverage of NGOs among community PWID, with NGO contact reducing injecting risk and increasing condom use and recruitment onto OAT and ART. We estimated the historic (1997–2021) and future (2022–2030, compared to no NGO activities from 2022) impact of NGOs in terms of the proportion of HIV/HCV infections averted and changes in HIV/HCV incidence. We estimated the future impact of scaling‐up NGOs to 80% coverage with/without scale‐up in OAT (5–20%) and ART (64–81%). We estimated the cost per disability‐adjusted life‐year (DALY) averted of current NGO provision over 2022–2041 compared to NGO activities stopping over 2022–2026, but restarting after that till 2041. We assumed average unit costs of US$80–90 per person‐year of NGO contact for PWID.

**Results:**

With existing coverage levels of NGOs, the model projects that NGOs have averted 20.0% (95% credibility interval: 13.3–26.1) and 9.6% (5.1–14.1) of new HIV and HCV infections among PWID over 1997–2021, respectively, and will avert 31.8% (19.6–39.9) and 13.7% (7.5–18.1) of HIV and HCV infections over 2022–2030. With NGO scale‐up, HIV and HCV incidence will decrease by 54.2% (43.3–63.8) and 30.2% (20.5–36.2) over 2022–2030, or 86.7% (82.9–89.3) and 39.8% (31.4–44.8) if OAT and ART are also scaled‐up. Without NGOs, HIV and HCV incidence will increase by 51.6% (23.6–76.3) and 13.4% (4.8–21.9) over 2022–2030. Current NGO provision over 2022–2026 will avert 102,736 (77,611–137,512) DALYs when tracked until 2041 (discounted 3% annually), and cost US$912 (702–1222) per DALY averted; cost‐effective at a willingness‐to‐pay threshold of US$1548/DALY averted (0.5xGDP).

**Conclusions:**

NGO activities have a crucial preventative impact among PWID in Ukraine which should be scaled‐up to help achieve HIV and HCV elimination. Disruptions could have a substantial detrimental impact.

## 1 INTRODUCTION

In Ukraine, most new HIV and hepatitis C virus (HCV) infections are attributable to injecting drug use (IDU) [1, 2], with there being a high prevalence of HIV (18% [3]) and HCV (58% seroprevalence [3]) among people who inject drugs (PWID).

Harm reduction interventions for PWID are a key component of Ukraine's national HIV strategy; mostly funded by the Global Fund [4]. This primarily funds non‐governmental organizations (NGOs), whose activities include distributing condoms and needles/syringes, providing HIV and HCV testing, and linkage to antiretroviral therapy (ART) and opioid agonist treatment (OAT). Our previous analyses of national data from five rounds of integrated bio‐behavioural assessment surveys (IBBA, 2009–2017) showed that contact with PWID‐targeted NGOs in Ukraine is associated with better preventive, HIV testing and HIV treatment outcomes [3]. These associations are heightened with longer contact with NGOs, suggesting a beneficial impact of NGOs [3].

The latest Global Fund grant for Ukraine incorporates a transition of funding to the government [5]. Due to competing economic priorities, there had been concerns that this would result in reductions in HIV funding. This had been exacerbated by the COVID‐19 pandemic, with the ongoing war with Russia making the provision of HIV programming uncertain [6]. Disruptions to HIV services following Russia's invasion in 2022 are likely to have increased HIV and HCV transmission among PWID, with data suggesting that ART initiations have reduced since the invasion [7] and that access to OAT reduced immediately following the invasion [8]. However, it is uncertain what impact these disruptions have had.

Considering these uncertainties, it is important to produce evidence for the effectiveness of existing intervention programming for guiding policymakers. We, therefore, use HIV and HCV transmission modelling to evaluate the impact and cost‐effectiveness of NGO activities for PWID in Ukraine. We also produce preliminary estimates of the impact of disruptions to services following Russia's invasion of Ukraine.

## 2 METHODS

### 2.1 Model description

A dynamic, deterministic HIV and HCV transmission model among the community and incarcerated PWID was developed (Figure 1), including stratifications for NGO and OAT status. The model is open with individuals entering due to initiating IDU and exiting through mortality from AIDS, HCV or other causes. The model tracks individuals following injecting cessation (ex‐PWID; which is modelled as permanent [9, 10, 11] with temporary cessation assumed to be part of the process of ongoing IDU) to capture HIV/HCV‐related mortality.

Figure 1Model Schematics. (a) Model schematic of initiation and cessation of injecting drug use (IDU), ageing and non‐disease‐related mortality. Drug‐related mortality is affected by OAT status (not shown). (b) Model schematic of HIV transmission, treatment and disease progression. (c) Model schematic of HCV transmission, treatment and disease progression. HCV disease progression and mortality rates elevated if HIV co‐infected [44, 45], but moderated if on ART [46]. (d) Model schematic of incarceration. (e) Model schematic of contact with non‐governmental organizations (NGO) and opiate agonist therapy (OAT). Abbreviations: ART, antiretroviral therapy; DC, decompensated cirrhosis; HCC, hepatocellular carcinoma; IDU, injecting drug use; LTFU, loss to follow‐up; PWID, people who inject drugs.

**Figure:**
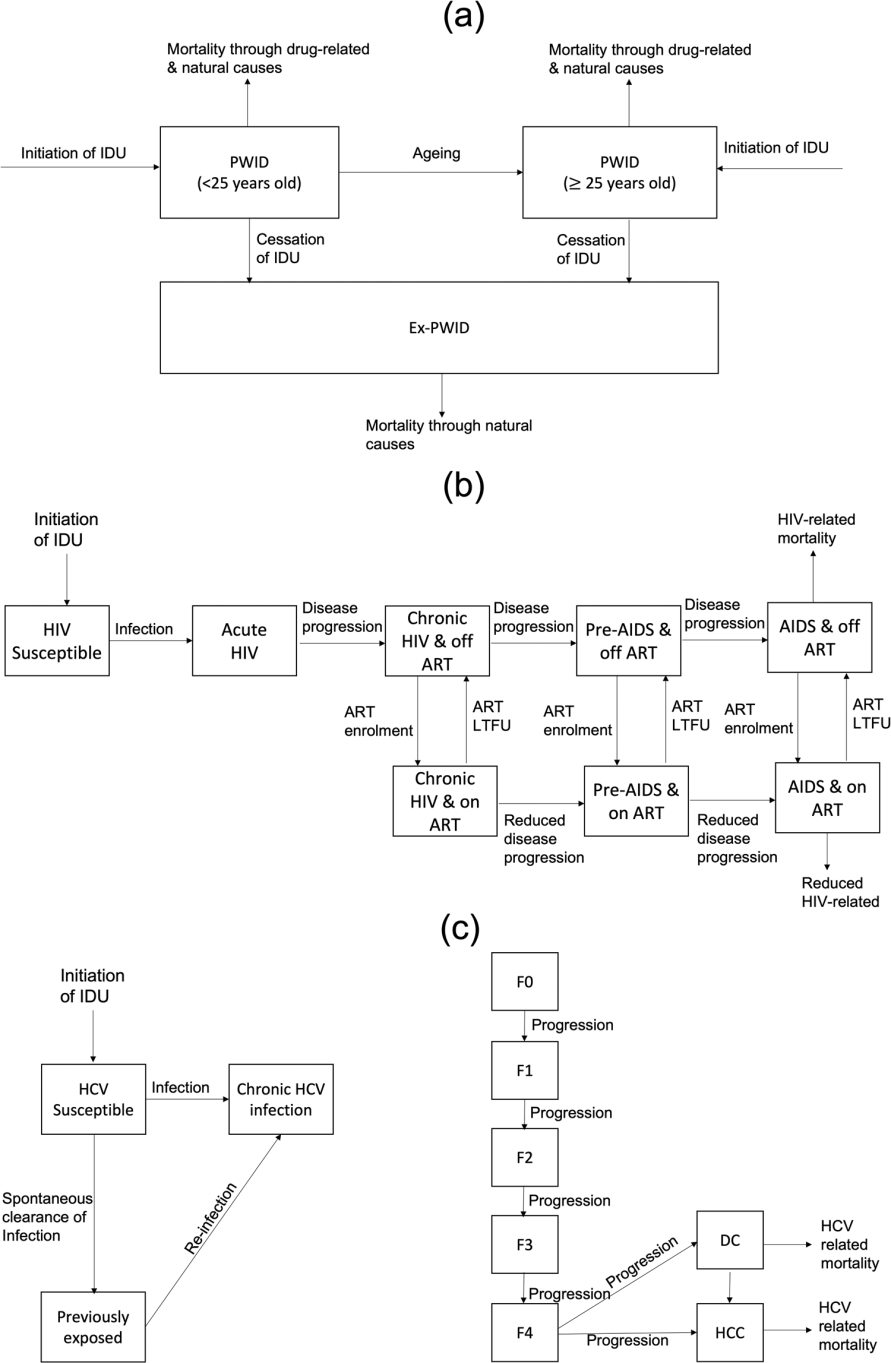

**Figure:**
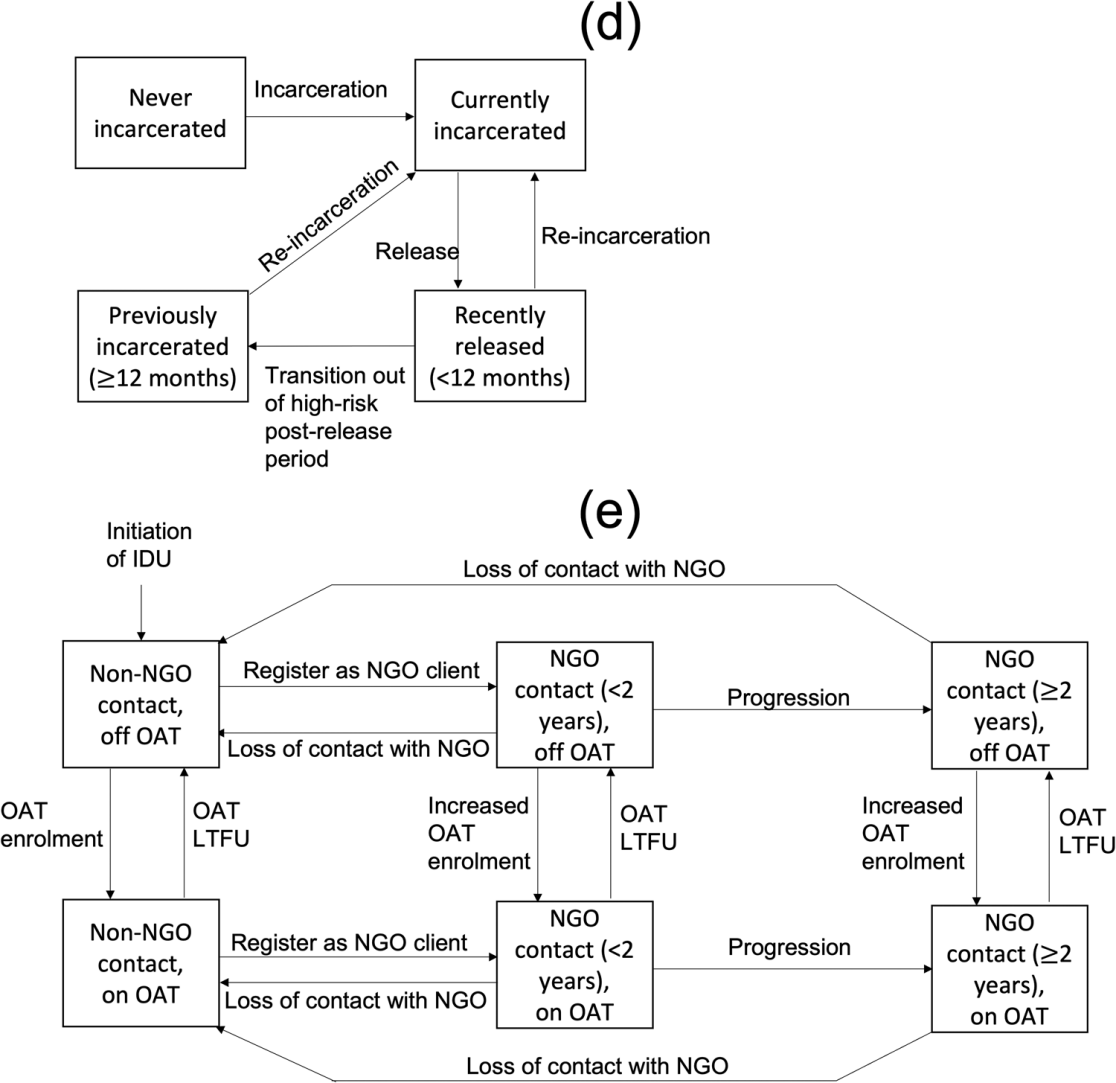

PWID enrol onto OAT at a time‐varying rate and leave OAT at fixed rates which differ by the current length of OAT. We assume excess mortality risk upon starting or leaving OAT [12], but reduced mortality on OAT otherwise [12]. PWID on OAT also have improved ART outcomes [13]. PWID are incarcerated and re‐incarcerated at differing rates, which depend on their age, gender and OAT status [14], but are released at a constant rate. PWID initiate contact with NGOs at rates depending upon their age and HIV status, and cease contact at a constant rate or upon incarceration. NGO clients have higher rates of OAT and ART initiation.

Susceptible PWID can acquire HIV and HCV through sharing of injecting equipment, with HIV also being sexually transmitted between PWID. Injecting transmission risk of HIV and HCV is lowered if on OAT [15, 16] or contact of NGOs, and is higher among female PWID and those previously incarcerated PWID (compared to never incarcerated PWID [17]). Injecting transmission risk among currently incarcerated PWID can be higher or lower than community PWID. ART reduces sexual and injecting HIV transmission [18]. Sexual HIV transmission risk depends upon the consistency of condom use (varies by age, gender, incarceration status, NGO status and OAT status) and the number of sexual contacts (varies by age, incarceration status, gender and OAT status). Sexual HIV transmission is modelled only between male and female PWID, with sexual transmission assumed negligible from other groups or in prison because <1% of male PWID report sex with men [19] and HIV prevalence is low in the general population.

Following HIV infection, individuals progress through different stages of HIV infection and can initiate ART as in Figure 1b [20]. Individuals with AIDS experience HIV‐related mortality and are assumed not to engage in HIV‐related risk behaviours unless they are receiving ART [20]. ART reduces HIV‐related mortality. PWID can be lost‐to‐care and then re‐enrolled onto ART at the same rate as ART‐naïve PWID.

Individuals exposed to HCV can either spontaneously clear their infection [21, 22] or can develop chronic HCV infection and HCV‐related disease as in Figure 1c. HCV treatment is not included in the model because existing low treatment levels (∼3000 PWID treated over 2016–2021) have had a negligible impact [23].

### 2.2 Model parameterization and calibration

The model is primarily parameterized using data from the 2011 (*n*=9069), 2013 (*n*=9502), 2015 (*n*=9405) and 2017 (*n*=10,076) national IBBA surveys [19, 24, 25, 26], using respondent‐driven sampling (RDS, 26–30 cities), and the 2014/5 Expanding Medication‐Assisted Therapy (ExMAT) bio‐behavioural survey (*n*=1612) [27], which used stratified sampling (five cities) of PWID currently/ever on OAT and never on OAT (using RDS). Table 1 summarizes key parameters and calibration data, with full details in Tables S1 and S2.

**Table 1:** Summary of main prior parameter ranges and calibration data (most recent estimates)

Parameter	Range	Source
**Calibration data**		
PWID population size	255,702–474,887	[47]
HIV prevalence among PWID	22.1–22.2%	2017 APH IBBA
HCV antibody prevalence among PWID	61.6–63.9%	2017 APH IBBA
Proportion of PWID in contact with NGOs	37.6–39.3%	2017 APH IBBA
Odds ratio of being in contact with NGO if HIV positive (vs. HIV negative)	2.00–2.23	2013/15/17 APH IBBA
Odds ratio of being in contact with NGO if <25 years old (vs. >=25 years old)	0.42–0.48	2013/15/17 APH IBBA
Proportion of PWID currently on OAT	4.4–5.3%	2017 APH IBBA
Odds ratio of being on OAT if in contact with NGOs	6.75–9.47	2015/17 APH IBBA
Proportion of HIV‐positive PWID on ART	35.3–47.2%	2017 APH IBBA
Odds ratio of being on ART if in contact with NGOs (vs. not in contact)	2.72–3.39	2015/17 APH IBBA
**Parameters**		
Average duration of injecting (years)	7.5–50	2011/13/15/17 APH IBBAs
Non‐disease‐related death rate among PWID (per 100 py)	1.99–7.14	[48]
Average length of each incarceration episode (months)	13–15	2011/13/15/17 APH IBBAs; EXMAT
Rate of loss to care from ART (per 100 py)	10.9–15.8	CPH HIV treatment database
Proportion of PWID on ART who are virally supressed	49–77%	[49]
Relative injecting risk (frequency of injecting with used equipment) if an NGO client versus not	0.55–0.97	2011/13/15/17 APH IBBAs
OR of using a condom if NGO client versus not	1.24–1.43	2011/13/15/17 APH IBBAs
Rate of loss to care from OAT if on OAT for <2 years (per year)	0.45–0.50	Estimated using data from [50]
Rate of loss to care from OAT if on OAT for >=2 years (per year)	0.1–0.15	
Relative risk of starting ART if on OAT versus not on OAT	1.50–2.33	[13]
Odds ratio of being virally supressed among those on ART if on OAT versus not on OAT	1.21–1.73	[13]
Relative risk of ART loss to care if on OAT versus not on OAT	0.63–0.95	[13]
Relative risk of HCV transmission through injecting if on OAT versus not on OAT	0.40–0.63	[15]
Relative risk of HIV transmission through injecting if on OAT versus not on OAT	0.32–0.67	[16]
Relative risk of incarceration if on OAT versus not on OAT	0.58–0.90	[14, 51]
Relative risk of non‐disease‐related mortality if on OAT versus not on OAT	0.28–0.39	[36]
Relative risk of non‐disease‐related mortality in first 4 weeks after starting OAT versus rest of time on OAT	0.94–4.10	[12]
Relative risk of non‐disease‐related mortality in first 4 weeks after leaving OAT versus rest of time off OAT	1.51–3.74	[12]

Note: Full details are in the Supplementary Materials.

Abbreviations: ART, antiretroviral therapy; NGOs, non‐governmental organizations; OAT, opioid agonist treatment; PWID, people who inject drugs.

We modelled the effect of NGOs on all PWID who self‐report being clients and/or those who report receiving sterile injecting equipment from them. In the model, NGO coverage begins in 1997, is scaled‐up to 37–49% coverage among community PWID by 2011 and is assumed stable thereafter [3]. Compared to other PWID, those in contact with NGOs are more likely to: be on OAT (aOR: 8.00, 95% CI: 6.75–9.47) and ART (if HIV positive, aOR: 3.03, 95% CI: 2.72–3.39), modelled as greater initiation rates among NGO contacts; use condoms (aOR: 1.33, 95% CI: 1.24–1.43); and have lower injecting risk (frequency injected with used equipment in last month, aIRR: 0.73, 95% CI: 0.55–0.97).

We calibrated the model (see Supplementary Materials) using an approximate Bayesian computation sequential Monte Carlo scheme [28] to various data including the: PWID population size; HIV and HCV seroprevalences and by age, gender and incarceration status; difference in HCV antibody prevalence by HIV status; proportion of PWID incarcerated in last year or ever; OAT and ART coverages and differences by NGO status; coverage of NGOs and differences by age and HIV status (Table 1); and proportion that have been contacted for <2 years (Table 1). This produced 1000 model fits which were used to give the median and 95% credibility intervals (95% CrI; 2.5th to 97.5th percentile range) for all model projections.

The goodness‐of‐fit was evaluated through the proportion of model runs that fall within at least one of the 95% confidence intervals of the HIV prevalence and HCV seroprevalence estimates for young and old male PWID, and young and old female PWID. Model fits were validated using the same goodness‐of‐fit metric for available HIV incidence data among all PWID (four estimates), or PWID in contact with NGO (four estimates) or not (one estimate).

### 2.3 Costs and health utilities

We adopted a funder's perspective to evaluate the cost‐effectiveness of NGO activities (funded by Global Fund) for PWID, including the costs of ART and OAT (funded by the Government). We searched published literature and reports for relevant unit costs for Ukraine, which were converted and inflated to 2018 USD (Table 2).

**Table 2:** Unit costs (in 2018 USD) and disability weights used in cost‐effectiveness analyses

Value	Value with uncertainty range	Source/comments
**Unit costs (per patient)**		
HIV‐negative PWID annual NGO cost	$90 (triangle $78–$101, accounts for regional variation in NSP cost, condoms and HCT are constant)	Includes cost of condoms, needle and syringe provision and HIV counselling and testing [29]
HIV‐positive PWID annual NGO cost	$80 (triangle $68–$91, accounts for regional variation in NSP cost, condoms)	Includes condoms and needle and syringe provision [29]
One off cost in first year of initiating ART if NGO contact	$132 (triangle $82–$182)	Cost of case management or psychosocial services for each person. Range is cost of case management to cost of psychosocial services, with the average of the two being used as the central estimate. Based on APH budget costs, we assume that 10% of NGO contacts initiating ART access case management or psychosocial services [29]
ART annual cost	$293.47 (triangle distribution $280.76–$312.53) [52]	Estimate of $276.50 includes drug, staff and test costs. We added 6% overheads and uncertainty bounds based on ART costs reported elsewhere [29]
OAT annual cost	$300 (triangle $194.78–$379.31)	Estimate of $300 includes drugs and provision, social support and incentives given to healthcare workers to support adherence. Uncertainty bounds based on OAT costs reported elsewhere [29]
**Disability weights**		
Acute or chronic HIV infection (on/off ART)	0.078 (triangle, 0.052–0.111)	No weights so used weights for HIV/AIDS: receiving antiretroviral treatment [30]
Pre‐AIDS, off ART	0.274 (triangle, 0.183–0.377)	Weights for HIV: symptomatic, pre‐AIDS [30]
AIDS, off ART	0.582 (triangle, 0.406–0.743)	Weights for AIDS: not receiving antiretroviral treatment [30]
Pre‐AIDS or AIDS, on ART	0.078 (triangle, 0.052–0.111)	Weights for HIV/AIDS: receiving antiretroviral treatment [30]
Compensated cirrhosis (F4)	0.114 (triangle, 0.078–0.159)	No weights, so used value for moderate abdominopelvic problem. Disability weights for F0–F4 are assumed to increase linearly from 0 for F0 [30]
Decompensated cirrhosis	0.178 (triangle, 0.1213–0.250)	Weights for decompensated liver cirrhosis [30]
Hepatocellular carcinoma	0.451 (triangle, 0.307–0.600)	No weights so used value for metastatic cancer [30]

Abbreviations: ART, antiretroviral therapy; NGOs, non‐governmental organizations; OAT, opioid agonist treatment; PWID, people who inject drugs.

NGO costs were obtained from a 2015 Ukraine costing study by Deloitte [29] and budgeting data from an NGO provider (Alliance for Public Health, APH). The budgeting data give the real‐life cost for existing provision but may underestimate overhead costs due to APH receiving funding from elsewhere. The Deloitte costs assumed a target coverage of 200 needles/syringes distributed annually per PWID reached, while APH distributes 130 assuming PWID also access needles/syringes from pharmacies. The baseline cost‐effectiveness analyses used Deloitte costs, with APH costs used in a sensitivity analysis.

Disability weights were taken from the 2013 Global Burden of Disease estimates [30], although HCV‐specific disability weights are not available. Therefore, as done previously [31, 32], disability weights for the moderate abdominopelvic problem were applied to compensated cirrhosis, assuming linear disability increase through fibrosis stages, and disability weights for metastatic cancer were applied to hepatocellular carcinoma. For coinfected individuals, disability weights were compounded multiplicatively.

### 2.4 Impact analysis

The calibrated model was first used to evaluate the impact of current levels of NGO provision over 1997–2021 or in 2021, compared to a counterfactual scenario which assumes all the differences observed among NGO contacts are intervention effects and turned off over 1997–2021. The impact was estimated in terms of new HIV and HCV infections prevented and differences in HCV and HIV incidence at end‐2021. By removing each NGO effect in turn over 1997–2021, we determined which effects contributed most to the infections averted.

We then evaluated the future impact on HIV and HCV incidence over 2022–2030 of continuing existing NGO provision or scaling‐up NGOs from 2022 to 80% coverage, with or without a concurrent scale‐up in OAT to 20% coverage and ART to the UNAIDS 90‐90‐90 targets (81% ART coverage and 90% viral suppression among those on ART). These were compared to a counterfactual where NGO provision ceased in 2022, with/without OAT and ART provision also ceasing in 2022. We also evaluated the impact of disruptions to services due to Russia's invasion. Based on Ukrainian data, we modelled a 26% reduction in ART initiations over March–November 2022 [7] and a 5% reduction in the coverage of OAT in March 2022 [8]. We assumed that NGO provision was affected similarly to OAT.

### 2.5 Cost‐effectiveness analysis

We estimated the cost‐effectiveness of ongoing NGO activities by comparing the status quo scenario (NGO coverage remains stable) up to 2041 with a counterfactual scenario in which all NGO activities (costs and benefits) cease for 5 years over 2022–2026 (“No NGO 2022–2026”), but then restart in 2027 and continue until 2041. All costs and utilities were discounted 3% annually. Incremental cost‐effectiveness ratios (ICERs) were estimated for each of the 1000 model fits as incremental costs divided by the incremental disability‐adjusted life‐years (DALYs) averted over 2022–2041. The median ICER was then compared to a willingness‐to‐pay threshold of 50% of Ukraine's GDP (US$1548) [33]. We also estimated the cost‐effectiveness of scaling‐up NGO coverage, by comparing with our status quo a scenario in which coverage increases to 80% over 2022–2026 and then returns to baseline levels after 2026.

We performed sensitivity analyses to test the effect of assumptions on the ICER. These included: incorporating APH costs ($28/year) for NGO contact (Baseline $80–$90/year); combining disability weights across HIV and HCV domains by taking the maximum value; changing the time horizon to 10/30 years (Baseline 20 years); changing annual discount rate to 0/5% (Baseline: 3%); incorporating costs for HCV disease‐related care (Assuming 0.14% of pre‐cirrhotics, 0.69% of compensated cirrhosis and 40% of decompensated cirrhosis access care, at yearly costs of $223, $316 and $631, respectively, based on Georgian data [43]); assume 50% of NGO contacts access psychosocial services/case management when initiating ART (Baseline 10%). We also investigated how the ICER would change if OAT and ART were scaled‐up to 20% and the UNAIDS 90‐90‐90 targets from 2022, respectively. We also considered how the ICER of scaling‐up NGOs would change if NGOs were 25% less effective when scaled‐up (equivalent to 50% lower effectiveness among new clients). Lastly, we estimated the minimum threshold proportion of the differences (in condom use, injecting risk, ART recruitment and OAT recruitment) observed among NGO contacts (vs. non‐NGO contacts) that needs to be an intervention effect for NGOs to be cost‐effective.

## 3 RESULTS

### 3.1 Baseline model projections

The calibrated model agrees well with HIV and HCV prevalence data (Figures S1 and S2), with 78.0% or 35.4% of model projections falling within at least one 95% CI of the HIV or HCV prevalence estimates by age and gender, respectively. The goodness‐of‐fit metric for HCV is lower primarily due to the large variability in the HCV prevalence estimates for old male PWID (varying between 55.2% and 65.8% over 2013–2017) despite each having overly precise 95% CI due to large sample sizes. Our calibration approach allows us to capture the overall uncertainty around these estimates with 81.3% of our model's HCV prevalence projections for old males falling within the overall data range over 2013–2017 (55.2–65.8%) despite only 39.7% of projections falling within at least one of the 95% CIs of the data estimates.

**Figure 2:**
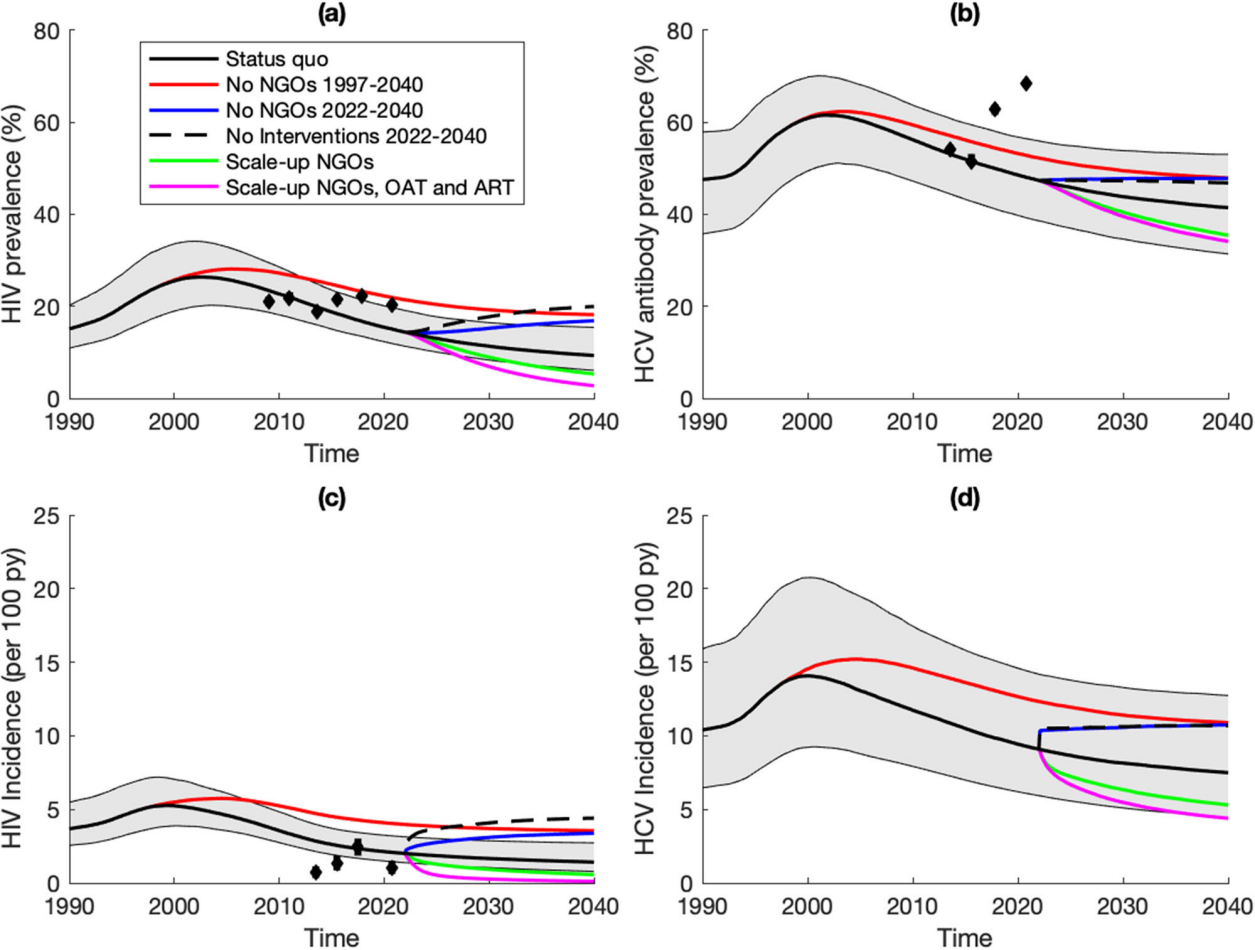
Model projections of (a) HIV prevalence, (b) HCV antibody prevalence, (c) HIV incidence and (d) HCV incidence. Notes: Solid black lines and grey shaded area show the median and 95% credibility interval of the baseline model fits with existing coverage levels of non‐governmental organizations (NGOs) continuing past 2022. Coloured lines show median model projections: without NGOs since 1997 (red); without NGOs from 2022 (blue); without NGOs, opioid agonist treatment (OAT) or antiretroviral therapy (ART) from 2022 (black dashed); with scaled‐up NGO coverage (60%) from 2022 (green); with scaled‐up NGO (60%) as well as OAT (20%) and ART (81%) coverages from 2022 (magenta). Data points with whiskers show data and their 95% confidence intervals with all datapoints based on community recruited people who inject drugs that were not used in model calibration.

The model also agrees well with available HIV incidence data (Figure 2 and Figure S3), which was not fit to, with 84.7% of model projections falling within at least one 95% CI of the incidence estimates for all PWID, or PWID in contact with NGO or not. The model projects HIV and HCV incidences of 2.0 per 100 py (95% CrI: 1.4–3.1) and 9.1 per 100 py (5.9–14.2) among all PWID in 2021, respectively. Projections suggest the HIV and HCV epidemics are decreasing. If existing interventions continue, HIV prevalence will decrease from 14.3% (11.3–18.9) in 2021 to 11.2% (8.3–16.3) in 2030, while chronic HCV prevalence will decrease from 41.0% (32.8–49.1) in 2021 to 37.5% (28.9–46.9) in 2030.

### 3.2 Existing impact of NGOs

**Figure 3:**
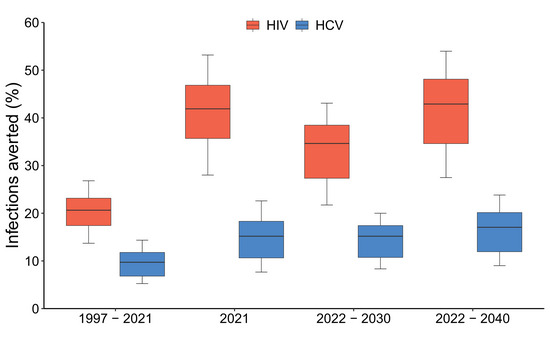
Proportion of new HIV and HCV infections averted among PWID by non‐governmental organizations (NGOs). Notes: Figure shows the impact NGOs have had since NGOs started (1997) and in the last year (2021) and the projected impact over 2022–2030 and 2022–2040. All are compared to no NGOs in past or future depending on the scenario. Boxes indicate the interquartile range, with the lines inside indicating the median impact, with whiskers representing 95% credibility intervals for the simulations.

**Figure 4:**
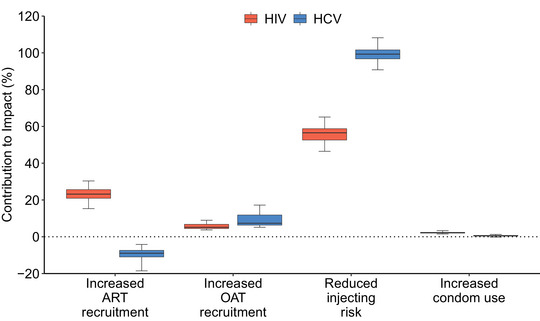
Contribution of each effect of non‐governmental organizations (NGOs) to the full impact of NGOs on HIV/HCV infections averted over 2022–2040. Notes: Boxes indicate the interquartile range, with the lines inside indicating the median impact, with whiskers representing 95% credibility intervals for the simulations. Abbreviations: ART, antiretroviral therapy; OAT, opioid agonist treatment.

In 2021, the model projects that 40.2% (32.0–46.0) of all PWID are in contact with NGOs, with HIV incidence being 28.5% (19.0–33.5) lower and HCV incidence 30.9% (20.8–36.4) lower among NGO contacts than non‐NGO contacts in the community. Without NGOs over 1997–2021, OAT and ART coverage in 2021 would have been 94.1% (80.6–99.7) and 47.9% (38.6–54.4) lower, respectively, while community PWID HIV and HCV incidence would have been 99.1% (52.7–154.1) and 35.2% (17.4–53.7) higher (Figure 2). In 2021, this translates to NGOs preventing 41.9% (28.0–53.2) and 15.2% (7.7–22.6) of new HIV and HCV infections (Figure 3), respectively, compared to the counterfactual. Figure 4 shows that the effect of NGOs on reducing injecting risk was the most important intervention effect for this impact in 2021, contributing 56.5% (46.5–65.1) and 99.3% (90.8–108.2) to the overall impact on averting HIV and HCV infections, respectively. For HIV, the effect of NGOs on increasing ART recruitment was also important, contributing 23.2% (15.3–30.4) to the overall impact of NGOs on averting HIV infections.

### 3.3 Future impact of NGOs

Continuing current pre‐invasion coverage levels of interventions, the model projects that HIV and HCV incidence will decrease by 19.7% (9.5–28.0) and 12.2% (5.3–17.5) over 2022–2030. This compares to HIV and HCV incidence increasing by 51.6% (23.6–76.3) and 13.4% (4.8–21.9) if there were no NGOs over 2022–2030 (Figure 2). This translates to NGOs preventing 31.8% (19.6–39.9) and 13.7% (7.5–18.1) of new HIV and HCV infections over 2022–2030 (Figure 3). If ART and OAT also ceased in 2022, HIV incidence would increase further, with a 64.7% (38.3–87.2) increase over 1 year or a doubling (100.2% increase, 95% CrI 56.5–140.3) in incidence over 2022–2030. Lastly, observed disruptions to OAT, ART and NGOs following Russia's invasion are projected to result in a small 1.3% (0.9–1.7) and 0.7% (0.4–0.9) increase in new HIV and HCV infections, respectively, over 2022, compared to if interventions had remained at pre‐invasion levels.

If the coverage of NGOs was increased from 2022 such that 80% of community PWID are in contact with NGOs by 2025, then HIV and HCV incidence would decrease by 54.2% (43.3–63.8) and 30.2% (20.5–36.2) over 2022–2030 (Figure 2). This scale‐up would increase the coverage of OAT and ART in 2030 by 79.9% (59.3–118.6) and 30.0% (21.1–45.4), with the coverage of OAT and ART reaching 8.0% (6.5–10.2) and 51.0% (44.3–57.8) by 2030, respectively.

Further reductions in incidence could be achieved if OAT and ART are scaled‐up to WHO/UNAIDS targets of 20% and 81% coverage, with HIV and HCV incidence reducing by 86.7% (82.9–89.3) and 39.8% (31.4–44.8) over 2022–2030.

### 3.4 Cost‐effectiveness of NGOs

Compared to a scenario without NGOs over 2022–2026, we estimated that the status quo scenario incurred a total incremental cost of US$94 million (75–121) over 2022–2041, with NGO, ART and OAT costs accounting for 78.4% (69.5–86.0), –1.7% (i.e. ART costs are on average saved in the status quo scenario; 95% CrI –5.5 to 3.3) and 23.4% (17.0–29.4) of these incremental costs, respectively. The status quo scenario would avert 102,736 (77,611–137,512) DALYs over 2022–2041, resulting in a median ICER of US$912 (702–1222) per DALY averted (Table 3). The ICER is cost‐effective, with 100% of model simulations being cost‐effective compared to the willingness‐to‐pay threshold of 50% of Ukraine's GDP (US$1548) [33] (Figures S4 and S5). We also find that compared to status quo coverage levels, scaling‐up NGO coverage over 2022–2026 is cost‐effective, with a median ICER of US$1204 (875–1602) per DALY averted (Table S3). NGO scale‐up would still be cost‐effective (ICER=US$1443, 95% CrI: 983–2077) if the effectiveness of NGOs is reduced by 25% when scaled‐up.

**Table 3:** Cost‐effectiveness of continuing current levels of non‐governmental organization (NGO) provision over 2022–2041 compared to a counterfactual where NGO activities cease over 2022–2026 and then continue afterwards

	No NGO 2022–2026	Status quo	Incremental
Cost of NGO (Million $; 2022–2041)	96 (74–122)	170 (132–214)	74 (58–92)
Cost of ART (Million $; 2022–2041)	146 (99–238)	145 (97–237)	–2 (–6 to 3)
Cost of OAT (Million $; 2022–2041)	40 (27–63)	62 (41–95)	22 (14–33)
Total costs (Million $; 2022–2041)	283 (223–390)	377 (303–503)	94 (75–121)
DALYs averted	—	—	102,736 (77,611–137,512)
Incremental cost‐effectiveness ratio (ICER; $ per DALY averted)	—	—	911.6 (702.4–1222.0)

Notes: Table shows the results using baseline cost assumptions and a discount rate of 3% per annum. Cells present median projections across 1000 model fits along with 95% credibility intervals in parentheses.

Abbreviations: ART, antiretroviral therapy; DALY, disability‐adjusted life year; OAT, opioid agonist treatment.

In sensitivity analyses (Figure S6), the ICER was most sensitive to reducing the time horizon to 10 years where it became not cost‐effective (ICER=US$2650/DALY averted). In all other sensitivity analyses, NGOs remained highly cost‐effective. In threshold analyses, NGOs remain cost‐effective if >60% of the observed differences between PWID that are NGO contacts and those that are not are due to intervention effects.

## 4 DISCUSSION

Model projections suggest that HIV and HCV incidence is 29–31% lower among NGO contacts than non‐NGO contacts, with recent NGO coverage levels (∼40%) averting 42% and 15% of new HIV and HCV infections in 2021. Over 2022–2030, if pre‐war coverage levels of NGOs were maintained, then HIV and HCV incidence would decrease by 20% and 12%, while it will decrease by 87% and 40% if NGO coverage is doubled alongside increasing the coverage of OAT (20%) and ART (81%). Conversely, rapid increases in incidence could occur if interventions are disrupted, with HIV and HCV incidence increasing by 52% and 13% over 2022–2030 if NGO activities stopped in 2022, and HIV incidence doubling if OAT and ART also ceased. Much smaller effects are projected with ongoing disruptions in services following Russia's invasion, with <2% additional new HIV or HCV infections in 2022. However, this does not count for any other changes that may have occurred due to the war. Importantly, existing NGO programming is cost‐effective (US$912/DALY averted), with this remaining robust under numerous sensitivity analyses including if NGOs were scaled up.

### 4.1 Strengths and limitations

A strength of our modelling includes the use of detailed data within a Bayesian framework for model parameterization and calibration, incorporating uncertainty into model projections. We also evaluated the multiple potential benefits of being an NGO contact and modelled their impact on HIV and HCV transmission. However, there are limitations. The IBBA surveys had limited data on younger PWID (<25 years), with this sub‐population thought to be under‐represented. To account for this, we primarily calibrated the model to data from older PWID and included additional uncertainty in estimates of the proportion of PWID <25 during model calibration. We also assumed that the associations with NGO contact identified in the IBBAs were causative, such that contacts of NGOs have lower injecting risk, increased condom use and improved recruitment onto ART and OAT. We think this is reasonable because NGOs undertake activities that aim to have these effects, including the provision of condoms and needles/syringes, HIV prevention education, HIV testing and counselling, and linkage to ART and OAT. Additionally, our prior analyses showed that associations with NGO contact increased with a longer duration of contact [3] adding further evidence for there being a causative effect. Importantly, our threshold analyses suggest only 60% of the observed differences between PWID with and without NGO contact needs to be an intervention effect for NGOs to be cost‐effective. Due to a lack of data, we were unable to assess the effects of NGOs on other outcomes, such as viral suppression, which may be improved for contacts receiving case management and psychosocial services when starting ART.

We did not incorporate the potential effects of the COVID‐19 pandemic, although data suggest no detrimental impact on NGO (increased from 171,743 unique contacts in 2019 to 201,443 in 2020) and OAT coverage levels [35]. It is too early to determine the detrimental effects of the ongoing Russian invasion. Although our results show that the closure of NGOs or the ceasing of OAT and ART provision could result in substantial health harm, preliminary national data suggest that interventions did not decrease to a large extent, with our modelling suggesting this may have only increased incidence marginally.

### 4.2 Comparisons with existing studies

Several previous modelling analyses for Ukraine have evaluated the impact and/or cost‐effectiveness of scaling‐up OAT [36, 37, 38, 39] and ART among PWID [40, 41]; finding that expanding OAT and ART could have a significant impact on HIV transmission and would be cost‐effective. However, only one study has modelled both HIV and HCV transmission, focusing on the impact of OAT on reducing mortality in Kyiv [36]. A previous analysis by our team also estimated the impact and cost‐effectiveness of an early NGO for PWID in Odessa over 1999–2000 [42], suggesting that this intervention was cost‐effective ($97/HIV infection averted in 1999 dollars) but unlikely to substantially reduce HIV prevalence. Our analyses add substantially to these previous analyses, by evaluating the historical, current and potential future impact and cost‐effectiveness of national NGO programming on both HIV and HCV transmission. Notably, our analysis captures the evolving role of NGOs in Ukraine, which in addition to providing condoms and syringes, also includes HIV testing and linkage to ART and OAT. Our analyses suggest the effects of NGOs, in particular reducing injecting risk and improving ART initiation, contribute significantly to reducing HIV transmission.

## 5 CONCLUSIONS

Our analyses suggest that PWID‐targeted NGOs in Ukraine are cost‐effective for controlling HIV and HCV transmission among PWID. Considering the ongoing transition in the funding of HIV services from the Global fund to the Ukrainian government [5], and possible disruptions that could occur due to the ongoing war, our analyses are important for showing that funding for PWID programming should continue because of the large impact these interventions are having. Indeed, our projections show that funding should be expanded to scale‐up NGO activities, OAT and HIV treatment to further decrease HIV and HCV transmission and so progress towards the WHO/UNAIDS targets of HIV and HCV elimination.

Our analyses also have implications for other settings, showing the important role and impacts that NGOs can have through not only providing needles/syringes, but also a package of services, including condom distribution and linkage to ART and OAT. Although the linkage of PWID to OAT by NGOs in Ukraine does not contribute substantially to their impact due to the low coverage of OAT (∼5%), this NGO effect could have more impact in other settings with more widespread provision of OAT. Also, given the high HIV prevalence among PWID in many EECA settings, it is likely that similar NGO interventions will be impactful and cost‐effective in other settings if they have comparable effectiveness. This should be investigated in other settings.

Insights from our analyses remain critical, especially with Russia's invasion of Ukraine. Findings suggest that ongoing disruptions to services since the war may have had a limited impact on HIV and HCV transmission. However, a considerable detrimental impact could occur with larger disruptions to NGO activities and other services. HIV incidence could increase by 65% within a year if interventions cease for PWID, with this detrimental impact increasing, the longer services are interrupted. This emphasizes that efforts to continue services for PWID have been crucial for maintaining gains achieved over recent years, and that such efforts must continue to ensure the protections of this vulnerable population.

## COMPETING INTERESTS

NS, TS, YS and OV work for the Alliance for Public Health (APH), Ukraine, which is a non‐governmental organization. The Global Fund to fight AIDS, tuberculosis, and malaria (GF) or other international funders had no role in these analyses or in decisions to publish. APH is one of the largest recipients in Ukraine of funding from the GF, and the salaries of YS and TS are funded through GF grants. JGW, FLA and PV report research grants from Gilead unrelated to this work.

## AUTHORS’ CONTRIBUTIONS

PV, JS and JGW developed the initial model aims and analysis plan with TS and OV. JS performed the model analyses. PV supported the modelling. SB and JGW gathered and analysed the cost data. FLA, NS, TS, YS and OV provided data. JS wrote the first draft of the manuscript. All authors contributed to the interpretation of model analyses, writing the manuscript and have approved the final version.

## FUNDING

This study was funded by Alliance for Public Health (APH), Ukraine, via the Global Fund to fight AIDS, tuberculosis, and malaria. .

## Supporting information

Supporting Information

## Data Availability

Model code will be made available following publication. The code will be shared with researchers who provide a methodologically sound proposal approved by JS and PV. Proposals should be directed to jack.stone@bristol.ac.uk and peter.vickerman@bristol.ac.uk; requesters will need to sign a data access agreement.
