# Supplementary material for: Modelling the impact and cost‐effectiveness of non‐governmental organizations on HIV and HCV transmission among people who inject drugs in Ukraine
Source: J Int AIDS Soc. 2023 Apr 3;26(4):e26073. doi: 10.1002/jia2.26073 (PMC10070931; doi:10.1002/jia2.26073)
Supplement: Supplementary file 1 — Supporting Information [file JIA2-26-e26073-s001.docx]

Supplementary Materials to: **Modelling the impact and cost-effectiveness of non-governmental organisations on HIV and HCV transmission among people who inject drugs in Ukraine**

Jack Stone, Adam Trickey, Josephine G Walker, Sandra Bivegete, Nadiya Semchuk, Yana Sazonova, Olga Varetska, Frederick L Altice, Tetiana Saliuk, Peter Vickerman

# Model Equations

The modelled population is defined as $X_{i,n,m,h,c,d}^{p,g,a}$, with

- p denoting injecting status: current injector (p=0), ex injector (p=1)
- g denoting gender: male (g=0), female (g=1)
- a denoting age: <25 years old (a=0), >= 25 years old (a=1)
- i denoting incarceration status: never incarcerated (i=0), currently incarcerated (i=1), previously incarcerated with last release within the last 12 months (i=2), previously incarcerated with last release not in the last 12 months (i=3).
- n denoting NGO contact status: not a contact (n=0), contact with < 2 years duration as a contact (n=1), contact with >= 2 years duration as a contact (n=2)
- m denoting OAT status: never on OAT (m=0), currently on OAT with <1 year duration of current episode (m=1), currently on OAT with $\geq$1 year duration of current episode (m=2), previously on OAT (m=3)
- h denoting HIV and ART status: susceptible (h=0), acute infection (h=1), chronic infection off ART (h=2), chronic infection on ART (h=3), Pre-AIDS off ART (h=4), Pre-AIDS on ART (h=5), AIDS off ART (h=6), AIDS on ART (h=7).
- c denoting HCV infection status: Susceptible (c=0), previously exposed (AB+ve, RNA-ve) (c=1), chronically infected (AB+ve, RNA+ve) (c=2)
- d denoting HCV disease stage: F0 (d=0), F1 (d=1), F2 (d=2), F3 (d=3), F4 (d=4), Decompensated cirrhosis (d=5), Hepatocellular carcinoma (d=6)

The model can be expressed as a set of ordinary differential equation

$\frac{dX_{i,n,m,h,c,d}^{p,g,a}}{\mathrm{dt}}=M_{i,n,m,h,c,d}^{p,g,a}+E_{i,n,m,h,c,d}^{p,g,a}+A_{i,n,m,h,c,d}^{p,g,a}+I_{i,n,m,h,c,d}^{p,g,a}+N_{i,n,m,h,c,d}^{p,g,a}+O_{i,n,m,h,c,d}^{p,g,a}+H_{i,n,m,h,c,d}^{p,g,a}+J_{i,n,m,h,c,d}^{p,g,a}+K_{i,n,m,h,c,d}^{p,g,a}+C_{i,n,m,h,c,d}^{p,g,a}+D_{i,n,m,h,c,d}^{p,g,a}$

Where

- $M_{i,n,m,h,c,d}^{p,g,a}$ denotes non-disease related mortality and cessation of injecting
- $E_{i,n,m,h,c,d}^{p,g,a}$ denotes entry into the model
- $A_{i,n,m,h,c,d}^{p,g,a}$ denotes ageing
- $I_{i,n,m,h,c,d}^{p,g,a}$ denotes movement between incarceration states
- $N_{i,n,m,h,c,d}^{p,g,a}$ denotes movement between the NGO states
- $O_{i,n,m,h,c,d}^{p,g,a}$ denotes movement between the OAT states
- $H_{i,n,m,h,c,d}^{p,g,a}$ denotes HIV infection
- $J_{i,n,m,h,c,d}^{p,g,a}$ denotes HIV disease progression
- $K_{i,n,m,h,c,d}^{p,g,a}$ denotes ART recruitment and loss to care
- $C_{i,n,m,h,c,d}^{p,g,a}$ denotes HCV infection
- $D_{i,n,m,h,c,d}^{p,g,a}$ denotes HCV disease progression

Each of these are described in detail below.

## Non-disease related mortality and cessation of injecting $\mathbf{(M}_{\mathbf{i,n,m,h,c,d}}^{\mathbf{p,g,a}}\mathbf{)}$

Upon ceasing injecting drugs, the model stops tracking age, incarceration status, NGO status and OAT status. Therefore, we sum over $a, i, n$ and $m$ in the third equation below.

$M_{i,n,m,h,c,d}^{0,g,a}=-\left( \mu_{0}+\mu_{c} \right)X_{i,n,m,h,c,d}^{0,g,a}$ for m=0,3 among current injectors (p=0)

$M_{i,n,m,h,c,d}^{0,g,a}=-\left( RR_{\mathrm{OAT}}^{\mu_{0}}\mu_{0}+\mu_{c} \right)X_{i,n,m,h,c,d}^{0,g,a}$ for m=1,2

$M_{0,0,0,h,c,d}^{1,g,0}=\mu_{c}\sum_{a} \sum_{i} \sum_{n} \sum_{m} X_{i,n,m,h,c,d}^{0,g,a}- \mu_{1}^{g}X_{0,0,0,h,c,d}^{1,g,0}$

Where,

- $\mu_{0}$ is the rate of non-disease related mortality among PWID (includes drug related death)
- $\mu_{c}$ is the rate of ceasing injecting drug use
- $RR_{\mathrm{OAT}}^{\mu_{0}}$ is the relative reduction in non-disease related mortality if PWID are on OAT
- $\mu_{1}^{g}$ is the rate of non-disease related mortality among ex-PWID which differs by gender ($g$).

## Entry into the model $\mathbf{(}\mathbf{E}_{\mathbf{i,n,m,h,c,d}}^{\mathbf{p,g,a}}\mathbf{)}$

Available PWID population size estimates for Ukraine suggest that the population size may have been higher in the past. We therefore model a decrease in the initiation of injecting over time. We assume that the number of PWID who initiate injecting decreases linearly between $yr_{\mathrm{change}}$ to $yr_{\mathrm{change}}+d_{change}$.

$E_{i,0,0,0,0,0}^{0,g,a}=p_{i}^{g,a}f\left( \mu_{0}+\mu_{c} \right)N_{0}$ for g=0,1; a=0,1; i=0,1,3

$E_{i,n,m,h,c,d}^{p,g,a}=0$ otherwise.

Where,

$$f=\left\{ \begin{aligned} 1 if t<yr_{\mathrm{change}} \\ 1-min(t-yr_{\mathrm{change}},d_{\mathrm{change}})&\Delta_{\mathrm{change}} if t\geq yr_{\mathrm{change}} \end{aligned} \right.$$

- $p_{i}^{g,a}$ is the proportion of new PWID that enter the model as gender $g$ with age $a$ and incarceration status $i$.
- $yr_{\mathrm{change}}$ is the date when the number of new PWID initiates per year begins to decrease.
- $d_{\mathrm{change}}$ is the length of time over which the number of new PWID initiates per year decreases.
- $\Delta_{\mathrm{change}}$ is the factor reduction in the number of new PWID initiates per year.
- $N_{0}$ is the size of the PWID population in 1985.

## Ageing$\mathbf{(}\mathbf{A}_{\mathbf{i,n,m,h,c,d}}^{\mathbf{p,g,a}}\mathbf{)}$

$A_{i,n,m,h,c,d}^{0,g,0}=-\zeta X_{i,n,m,h,c,d}^{0,g,0}$

$A_{i,n,m,h,c,d}^{0,g,1}=\zeta X_{i,n,m,h,c,d}^{0,g,0}$

$$A_{i,n,m,h,c,d}^{1,g,a}=0$$

Where,

$$\zeta=\frac{1}{25-\zeta_{0}}$$

$\zeta_{0}$ is the average age PWID start injecting among those that start injecting before the age of 25.

## Movement between incarceration states $\mathbf{(}\mathbf{I}_{\mathbf{i,n,m,h,c,d}}^{\mathbf{p,g,a}}\mathbf{)}$

$I_{0,n,m,h,c,d}^{0,g,a}=-\gamma_{0,m}^{g,a}X_{0,n,m,h,c,d}^{0,g,a}$

$I_{1,0,0,h,c,d}^{0,g,a}=-\tau X_{1,0,0,h,c,d}^{0,g,a}+\sum_{n} \left( \gamma_{0,0}^{g,a}X_{0,n,0,h,c,d}^{0,g,a}+\gamma_{1,0}^{g,a}X_{2,n,0,h,c,d}^{0,g,a}+\gamma_{1,0}^{g,a}X_{3,n,0,h,c,d}^{0,g,a} \right)$

$I_{1,0,3,h,c,d}^{0,g,a}=-\tau X_{1,0,3,h,c,d}^{0,g,a}+\sum_{n} \left( \gamma_{0,3}^{g,a}X_{0,n,3,h,c,d}^{0,g,a}+\gamma_{1,3}^{g,a}X_{2,n,3,h,c,d}^{0,g,a}+\gamma_{1,3}^{g,a}X_{3,n,3,h,c,d}^{0,g,a} \right)+ \left( 1-\frac{\left( \epsilon_{1}-1 \right)4\mu_{0}}{52} \right)\sum_{m=1,2} \sum_{n} \left( \gamma_{0,m}^{g,a}X_{0,n,m,h,c,d}^{0,g,a}+\gamma_{1,m}^{g,a}X_{2,n,m,h,c,d}^{0,g,a}+\gamma_{1,m}^{g,a}X_{3,n,m,h,c,d}^{0,g,a} \right)$

$I_{2,n,m,h,c,d}^{0,g,a}=\tau X_{1,n,m,h,c,d}^{0,g,a}-(\gamma_{1,m}^{g,a}+1)X_{2,n,m,h,c,d}^{0,g,a}$

$I_{3,n,m,h,c,d}^{0,g,a}=X_{2,n,m,h,c,d}^{0,g,a}-\gamma_{1,m}^{g,a}X_{3,n,m,h,c,d}^{0,g,a}$

$I_{i,n,m,h,c,d}^{1,g,a}=0$

Where,

- $\gamma_{0,m}^{g,a}$ is the incarceration rate for PWID of gender $g$, age group $a$, and OAT status $m$.
- $\gamma_{1,m}^{g,a}$ is the re-incarceration rate for PWID of gender $g$, age group $a$, and OAT status $m$.
- $1/\tau$ is the average length of each incarceration (in years).
- $\epsilon_{1}$ is the relative risk of all-cause mortality in the first four weeks after leaving OAT

## Movement between the NGO states $\mathbf{(N}_{\mathbf{i,n,m,h,c,d}}^{\mathbf{p,g,a}}\mathbf{)}$

$N_{i,0,m,h,c,d}^{0,g,a}=-\kappa_{h}^{a}X_{i,0,m,h,c,d}^{0,g,a}+\delta_{NGO}{(X}_{i,1,m,h,c,d}^{0,g,a}+X_{i,2,m,h,c,d}^{0,g,a})$ for i=0,2,3

$N_{i,1,m,h,c,d}^{0,g,a}=\kappa_{h}^{a}X_{i,0,m,h,c,d}^{0,g,a}-(\delta_{NGO}+\frac{1}{2})X_{i,1,m,h,c,d}^{0,g,a}$ for i=0,2,3

$N_{i,2,m,h,c,d}^{0,g,a}=\frac{1}{2}X_{i,1,m,h,c,d}^{0,g,a}-\delta_{NGO}X_{i,2,m,h,c,d}^{0,g,a}$ for i=0,2,3

$N_{1,n,m,h,c,d}^{0,g,a}=0$

$N_{i,n,m,h,c,d}^{1,g,a}=0$

Where

- $\kappa_{h}^{a}$ is the rate PWID become NGO contacts which varies by age group $a$ and HIV infection status $h$
- $\delta_{NGO}$ is the rate of loss of NGO contact

## Movement between the OAT states $\left( \mathbf{O}_{\mathbf{i,n,m,h,c,d}}^{\mathbf{p,g,a}} \right)$

$O_{i,n,0,h,c,d}^{0,g,a}=-l_{n}X_{i,n,0,h,c,d}^{0,g,a}$ for i=0,2,3

$O_{i,n,1,h,c,d}^{0,g,a}=(1-\frac{\left( \epsilon_{0}-1 \right)4\mu_{0}}{52})l_{n}(X_{i,n,0,h,c,d}^{0,g,a}+ X_{i,n,2,h,c,d}^{0,g,a})-(1+ \delta_{OAT}^{1})X_{i,n,1,h,c,d}^{0,g,a}$ for i=0,2,3

$O_{i,n,2,h,c,d}^{0,g,a}=X_{i,n,1,h,c,d}^{0,g,a}-\delta_{OAT}^{2}X_{i,n,2,h,c,d}^{0,g,a}$ for i=0,2,3

$O_{i,n,3,h,c,d}^{0,g,a}=- l_{n}X_{i,n,3,h,c,d}^{0,g,a}+ {(1-\frac{\left( \epsilon_{1}-1 \right)4\mu_{0}}{52})(\delta}_{OAT}^{1}X_{i,n,1,h,c,d}^{0,g,a}+ \delta_{OAT}^{2}X_{i,n,2,h,c,d}^{0,g,a})$ for i=0,2,3

$$O_{1,n,m,h,c,d}^{0,g,a}=0$$

$$O_{i,n,m,h,c,d}^{1,g,a}=0$$

Where,

- $l_{n}$ is the rate that PWID initiate OAT which varies by their NGO status $n$.
- $\delta_{OAT}^{m}$ is the rate that PWID cease OAT which varies by their current length of OAT $m ($m=1: <1 year of OAT; $m=2$: $\geq$1 year of OAT).
- $\epsilon_{0}$ is the relative risk of all-cause mortality in the first four weeks after starting OAT.
- $\epsilon_{1}$ is the relative risk of all-cause mortality in the first four weeks after leaving OAT.

## HIV infection $\mathbf{(H}_{\mathbf{i,n,m,h,c,d}}^{\mathbf{p,g,a}}\mathbf{)}$

$$H_{i,n,m,0,c,d}^{p,g,a}={-{(\Lambda}_{i,n,m}^{p,g,a}+\bar{\Lambda}}_{i,n,m}^{p,g,a}) X_{i,n,m,0,c,d}^{p,g,a}$$

$$H_{i,n,m,1,c,d}^{p,g,a}={{(\Lambda}_{i,n,m}^{p,g,a}+\bar{\Lambda}}_{i,n,m}^{p,g,a}) X_{i,n,m,0,c,d}^{p,g,a}$$

Where,

- $\Lambda_{i,n,m}^{p,g,a}$ is the HIV force of infection through IDU
- $\bar{\Lambda}_{i,n,m}^{p,g,a}$is the force of infection through sexual transmission

## HIV force of infection through IDU $\boldsymbol{(\Lambda}_{\mathbf{i,n,m,}}^{\mathbf{p,g,a}}\mathbf{)}$

For PWID in prison $(i=1)$

$$\Lambda_{1,n,m}^{0,g,a}=\lambda_{HIV}\eta_{1,n,m}^{g}\frac{\sum_{g} \sum_{a} \sum_{n} \sum_{m} \sum_{c} \sum_{d} (\eta_{1,n,m}^{g}Q_{1,n,m}^{g,a})}{\sum_{g} \sum_{a} \sum_{n} \sum_{m} \sum_{c} \sum_{d} \sum_{h\neq6} (\eta_{1,n,m}^{g}X_{1,n,m,h,c,d}^{0,g,a})}$$

For PWID in the community $\left( i\neq1 \right)$ who are<25$(a=0)$

$$\Lambda_{i,n,m}^{0,g,0}={\lambda_{HIV}\eta}_{i,n,m}^{g}\left[ \Psi\frac{\sum_{i\neq1} \sum_{g} \sum_{n} \sum_{m} \sum_{c} \sum_{d} \left( \eta_{i,n,m}^{g}Q_{i,n,m}^{g,0} \right)}{\sum_{i\neq1} \sum_{g} \sum_{n} \sum_{m} \sum_{c} \sum_{d} \sum_{h\neq6} (\eta_{i,n,m}^{g}X_{i,n,m,h,c,d}^{0,g,0})}+\left( 1-\Psi\right)\frac{\sum_{i\neq1} \sum_{g} \sum_{n} \sum_{m} \sum_{c} \sum_{d} \sum_{a} \left( \eta_{i,n,m}^{g}Q_{i,n,m}^{g,a} \right)}{\sum_{i\neq1} \sum_{g} \sum_{n} \sum_{m} \sum_{c} \sum_{d} \sum_{h\neq6} \sum_{a} (\eta_{i,n,m}^{g}X_{i,n,m,h,c,d}^{0,g,a})} \right]$$

For PWID in the community $\left( i\neq1 \right)$ who are $\geq$25$(a=1)$

$$\Lambda_{i,n,m}^{0,g,1}={\lambda_{HIV}\eta}_{i,n,m}^{g}\left[ \Psi\frac{\sum_{i\neq1} \sum_{g} \sum_{n} \sum_{m} \sum_{c} \sum_{d} \left( \eta_{i,n,m}^{g}Q_{i,n,m}^{g,1} \right)}{\sum_{i\neq1} \sum_{g} \sum_{n} \sum_{m} \sum_{c} \sum_{d} \sum_{h\neq6} (\eta_{i,n,m}^{g}X_{i,n,m,h,c,d}^{0,g,1})}+\left( 1-\Psi\right)\frac{\sum_{i\neq1} \sum_{g} \sum_{n} \sum_{m} \sum_{c} \sum_{d} \sum_{a} \left( \eta_{i,n,m}^{g}Q_{i,n,m}^{g,a} \right)}{\sum_{i\neq1} \sum_{g} \sum_{n} \sum_{m} \sum_{c} \sum_{d} \sum_{h\neq6} \sum_{a} (\eta_{i,n,m}^{g}X_{i,n,m,h,c,d}^{0,g,a})} \right]$$

$$For ex-PWID \left( p=1 \right),$$

$$\Lambda_{i,n,m}^{1,g,0}=0$$

Where,

$$Q_{i,n,m}^{g,a}=\alpha_{1}X_{i,n,m,1,c,d}^{0,g,a}+X_{i,n,m,2,c,d}^{0,g,a}+\alpha_{3}X_{i,n,m,4,c,d}^{0,g,a}+z_{m}(X_{i,n,m,3,c,d}^{0,g,a}+\alpha_{3}X_{i,n,m,5,c,d}^{0,g,a}+\alpha_{3}X_{i,n,m,7,c,d}^{0,g,a})$$

and

- $\lambda_{HIV}$ is the HIV transmission rate through IDU
- $\eta_{i,n,m}^{g}$is the HIV injecting transmission risk of PWID relative to PWID who are male (g=0), have never been incarcerated (i=0), are not an NGO contact (n=0), and are not on OAT (m=0). By definition, $\eta_{0,0,0}^{0}=1$
- $\Psi$ is the amount of assortative mixing by age. Otherwise mixing is random.
- $\alpha_{1}$ is the relative increase in HIV transmissibility in the acute stage of infection compared to the latent stage of infection
- $\alpha_{3}$ is the relative increase in HIV transmissibility in the pre-AIDS and AIDS stages of infection compared to the latent stage of infection
- $z_{m}$ is the relative reduction in HIV transmissibility for PWID on ART which varies by OAT status $(m)$

## HIV force of infection through sexual transmission ${\mathbf{(}\bar{\boldsymbol{\Lambda}}}_{\mathbf{i,n,m}}^{\mathbf{p,g,a}}\mathbf{)}$

The model includes sexual transmission between male and female PWID in the community only. No sexual HIV transmission is assumed from other groups or in prison because little sex between men is reported by PWID (<=1%) and HIV prevalence is low in the general population.

Let:

- $m_{i,n,m}^{g,a}$ be the proportion of PWID who are sexually active, which differs by gender $g$, age group $a$, incarceration status $i$, NGO status $n$, and OAT status $m$.
- $s_{i,n,m}^{g,a}$be the average number of sexual contacts among sexually active PWID of gender $g$, age group $a$, incarceration status $i$, NGO status $n$, and OAT status $m$
- $L^{g}$ be the proportion of PWID’s sexual partners that are also PWID, which varies by gender.
- $N_{i,n,m}^{g,a}$ be the number of PWID of gender $g$, age group $a$, incarceration status $i$, NGO status $n$, and OAT status $m$

We assume no sexual contacts in prison, such that $m_{i,n,m}^{g,a}$ and $s_{i,n,m}^{g,a}$ are 0 for $i=1$.

Then the probability, $r_{i,n,m}^{g,a}$, of mixing with a PWID of gender $g$, age group $a$, incarceration status $i$, NGO status $n$, and OAT status $m$ is proportional to the number of sexual contacts they provide and is given by:

$$r_{i,n,m}^{g,a}=\frac{m_{i,n,m}^{g,a}s_{i,n,m}^{g,a}N_{i,n,m}^{g,a}}{\sum_{i'} \sum_{a'} \sum_{m'} \sum_{n'} m_{i',n',m'}^{g',a'}s_{i',n',m'}^{g',a'}N_{i',n',m'}^{g',a'}}$$

We allow the sexual behaviours of males with females to determine who females have sex with. The total number of PWID partners that male PWID have with female PWID in age group $a$, incarceration status $i$, NGO status $n$, and OAT status $m$ is given by:

$$r_{i,n,m}^{1,a}\sum_{a} \sum_{i} \sum_{n} \sum_{m} L^{0}m_{i,n,m}^{0,a}s_{i,n,m}^{0,a}N_{i,n,m}^{0,a}$$

Thus the total number of PWID partners that each female PWID in age group $a$, incarceration status $i$, NGO status $n$, and OAT status $m$ has is given by:

$$\frac{r_{i,n,m}^{1,a}}{N_{i,n,m}^{1,a}}\sum_{a} \sum_{i} \sum_{n} \sum_{m} L^{0}m_{i,n,m}^{0,a}s_{i,n,m}^{0,a}N_{i,n,m}^{0,a}$$

And so the total number of male PWID partners of age group $\hat{a}$, incarceration status $\hat{i}$, NGO contact $\hat{n}$, and OAT status $\hat{m}$ that females in age group $a$ incarceration status $i$, NGO contact $n$, and OAT status $m$ have is given by:

$$r_{\hat{i},\hat{n},\hat{m}}^{0,\hat{a}}\frac{r_{i,n,m}^{1,a}}{N_{i,n,m}^{1,a}}\sum_{a} \sum_{i} \sum_{n} \sum_{m} L^{0}m_{i,n,m}^{0,a}s_{i,n,m}^{0,a}N_{i,n,m}^{0,a}$$

Substituting the expression for $r_{\hat{i}}^{0,\hat{a}}$ simplifies this expression to:

$$\frac{r_{i,n,m}^{1,a}}{N_{i,n,m}^{1,a}}L^{0}m_{\hat{i},\hat{n},\hat{m}}^{0,\hat{a}}s_{\hat{i},\hat{n},\hat{m}}^{0,\hat{a}}N_{\hat{i},\hat{n},\hat{m}}^{0,\hat{a}}$$

The force of infection ($\bar{\Lambda}_{i,n,m}^{p,g,a}$) is then given by:

For a susceptible male PWID (p=0 and i$\neq$1):

$$\bar{\Lambda}_{i,n,m}^{0,0,a}={m_{i,n,m}^{0,a}s_{i,n,m}^{0,a}\lambda}^{0}L^{0}\left[ \sum_{a^{'}} \sum_{i^{'}} \sum_{n^{'}} \sum_{m^{'}} \left( 1-\zeta_{c}C_{1,a^{'},i^{'},n^{'},m^{'}}^{0,a,i,n,m} \right)r_{i^{'},n^{'},m^{'}}^{1,a^{'}}B_{i^{'},n^{'},m^{'}}^{1,a^{'}} \right]$$

For a susceptible female PWID (p=0 and $i\neq$1):

$$\bar{\Lambda}_{i,n,m}^{0,1,a}=\lambda^{1}\left( \left[ \sum_{a^{'}} \sum_{i^{'}} \sum_{n^{'}} \sum_{m^{'}} \left( 1-\zeta_{c}C_{{0,a}^{'},i^{'},n^{'},m^{'}}^{1,a,i,n,m} \right)\frac{r_{i,n,m}^{1,a}}{N_{i,n,m}^{1,a}}L^{0}m_{i^{'},n^{'},m^{'}}^{0,a^{'}}s_{i^{'},n^{'},m^{'}}^{0,a^{'}}N_{i^{'},n^{'},m^{'}}^{0,a^{'}}B_{i^{'},n^{'},m^{'}}^{1,a^{'}} \right] \right)$$

For male ex-PWID:

$\bar{\Lambda}_{i,n,m}^{1,0,a}=\bar{\Lambda}_{i,n,m}^{0,0,a}$

For female ex-PWID

$\bar{\Lambda}_{i,n,m}^{1,1,a}=\bar{\Lambda}_{i,n,m}^{0,1,a}$

Where

- $B_{i,n,m}^{g,a}$ is the weighted (to account for different transmission risks by HIV stage and ART status) HIV prevalence among PWID of gender $g$, age group $a$ incarceration status $i$, NGO status $n$, and OAT status $m$ and is given by:
- $B_{i,n,m}^{g,a}=\frac{\alpha_{1}X_{i,n,m,1,c,d}^{0,g,a}+X_{i,n,m,2,c,d}^{0,g,a}+\alpha_{3}X_{i,n,m,4,c,d}^{0,g,a}+z_{m}(X_{i,n,m,3,c,d}^{0,g,a}+\alpha_{3}X_{i,n,m,5,c,d}^{0,g,a}+\alpha_{3}X_{i,n,m,7,c,d}^{0,g,a})}{\sum_{h\neq6} X_{i,n,m,h,c,d}^{g,a}}$
- $\zeta_{c}$is the efficacy of condom use at reducing HIV sexual transmission
- $C^{g,a,i,n,m}$ is the condom use among PWID of gender $g$, age group $a,$ incarceration status $i$, NGO status $n$, and OAT status $m$
- $C_{g',a^{'},i^{'},n^{'},m^{'}}^{g,a,i,n,m}=\frac{C^{g,a,i,n,m}+C^{g',a',i',n',m'}}{2}$ is the average condom use between PWID of gender $g$, age group $a,$ incarceration status $i$, NGO status $n$, and OAT status $m$ and PWID of gender $g'$, age group $a^{'},$ incarceration status $i'$, NGO status $n'$, and OAT status $m$’
- $\lambda^{g}$ is the risk of acquiring HIV through sexual transmission

## Relative reduction in HIV transmissibility for PWID on ART$\boldsymbol{(}\boldsymbol{z}_{\boldsymbol{m}}\boldsymbol{)}$

Let $V_{m}$ denote the proportion of PWID on ART who are virally supressed which depends on OAT status $m$. To determine the decrease in HIV transmission risk among virally suppressed and unsuppressed PWID on ART, we estimated the log difference between the baseline plasma viral load ($PVL-v_{b})$ for PWID off ART, and PWID on ART with suppressed ($v_{s})$ PVL $\Delta_{s}=v_{b}-v_{s}$, or unsuppressed ($v_{u})$ PVL$\Delta_{u}={v_{b}-v}_{u}$. Because prior studies (1, 2) suggest HIV transmission risk increases (factor $r_{t}$) for each log increase in PVL, these log differences in PVL were used to estimate the relative decrease in transmission risk among virally suppressed ${(e}_{s})$ and unsuppressed ${(e}_{u})$ PWID on ART:

$$\begin{aligned} e_{s}=1/{(r}_{t}^{\Delta_{s}}) \\ e_{u}=1/{(r}_{t}^{\Delta_{u}}) \end{aligned}$$

The average reduction in HIV transmissibility for PWID off OAT $(m=0,3)$ or on OAT $(m=1,2)$ is then given by

$$z_{m}=V_{m}e_{s}+\left( 1-V_{m} \right)e_{u}$$

## HIV disease progression($\mathbf{J}_{\mathbf{i,n,m,h,c,d}}^{\mathbf{p,g,a}}\mathbf{)}$

$$J_{i,n,m,1,c,d}^{p,g,a}=-h_{a}X_{i,n,m,1,c,d}^{p,g,a}$$

$J_{i,n,m,2,c,d}^{p,g,a}=h_{a}X_{i,n,m,1,c,d}^{p,g,a}-h_{c}X_{i,n,m,2,c,d}^{p,g,a}$

$$J_{i,n,m,3,c,d}^{p,g,a}=h_{a}X_{i,n,m,1,c,d}^{p,g,a}-\mathrm{Ah}_{c}X_{i,n,m,3,c,d}^{p,g,a}$$

$$J_{i,n,m,4,c,d}^{p,g,a}=h_{c}X_{i,n,m,2,c,d}^{p,g,a}-h_{p}X_{i,n,m,4,c,d}^{p,g,a}$$

$$J_{i,n,m,5,c,d}^{p,g,a}=\mathrm{Ah}_{c}X_{i,n,m,3,c,d}^{p,g,a}-Ah_{p}X_{i,n,m,5,c,d}^{p,g,a}$$

$$J_{i,n,m,6,c,d}^{p,g,a}=h_{p}X_{i,n,m,4,c,d}^{p,g,a}-\mu_{A}X_{i,n,m,6,c,d}^{p,g,a}$$

$$J_{i,n,m,7,c,d}^{p,g,a}=Ah_{p}X_{i,n,m,5,c,d}^{p,g,a}-{A\mu}_{A}X_{i,n,m,7,c,d}^{p,g,a}$$

Where

- $\frac{1}{h_{a}}$is the average duration of the acute stage of HIV infection
- $\frac{1}{h_{c}}$is the average duration of the chronic stage of HIV infection, in the absence of ART
- $\frac{1}{h_{p}}$is the average duration of the pre-AIDS stage of HIV infection, in the absence of ART
- A is the relative reduction in HIV progression and mortality if on ART
- $\mu_{A}$ is the AIDS mortality rate in the absence of ART

## ART recruitment and loss to care ($\mathbf{K}_{\mathbf{i,n,m,h,c,d}}^{\mathbf{p,g,a}}\mathbf{)}$

$K_{i,n,m,h,c,d}^{p,g,a}=0$ if $h=0,1$

$$K_{i,n,m,2,c,d}^{0,g,a}={-\Gamma}_{n,m}^{a}X_{i,n,m,2,c,d}^{0,g,a}+\delta_{ART}^{m}X_{i,n,m,3,c,d}^{0,g,a}$$

$$K_{i,n,m,3,c,d}^{0,g,a}=\Gamma_{n,m}^{a}X_{i,n,m,2,c,d}^{0,g,a}-\delta_{ART}^{m}X_{i,n,m,3,c,d}^{0,g,a}$$

$$K_{i,n,m,4,c,d}^{0,g,a}={-\Gamma}_{n,m}^{a}X_{i,n,m,4,c,d}^{0,g,a}+\delta_{ART}^{m}X_{i,n,m,5,c,d}^{0,g,a}$$

$$K_{i,n,m,5,c,d}^{0,g,a}=\Gamma_{n,m}^{a}X_{i,n,m,4,c,d}^{0,g,a}-\delta_{ART}^{m}X_{i,n,m,5,c,d}^{0,g,a}$$

$$K_{i,n,m,6,c,d}^{0,g,a}={-\Gamma}_{n,m}^{a}X_{i,n,m,6,c,d}^{0,g,a}+\delta_{ART}^{m}X_{i,n,m,7,c,d}^{0,g,a}$$

$$K_{i,n,m,7,c,d}^{0,g,a}=\Gamma_{n,m}^{a}X_{i,n,m,6,c,d}^{0,g,a}-\delta_{ART}^{m}X_{i,n,m,7,c,d}^{0,g,a}$$

$$K_{i,n,m,2,c,d}^{1,g,a}={-\Gamma}_{1,1}^{a}X_{i,n,m,2,c,d}^{1,g,a}+\delta_{ART}^{1}X_{i,n,m,3,c,d}^{1,g,a}$$

$$K_{i,n,m,3,c,d}^{1,g,a}=\Gamma_{1,1}^{a}X_{i,n,m,2,c,d}^{1,g,a}-\delta_{ART}^{1}X_{i,n,m,3,c,d}^{1,g,a}$$

$$K_{i,n,m,4,c,d}^{1,g,a}={-\Gamma}_{1,1}^{a}X_{i,n,m,4,c,d}^{1,g,a}+\delta_{ART}^{1}X_{i,n,m,5,c,d}^{1,g,a}$$

$$K_{i,n,m,5,c,d}^{1,g,a}=\Gamma_{1,1}^{a}X_{i,n,m,4,c,d}^{1,g,a}-\delta_{ART}^{1}X_{i,n,m,5,c,d}^{1,g,a}$$

$$K_{i,n,m,6,c,d}^{1,g,a}={-\Gamma}_{1,1}^{a}X_{i,n,m,6,c,d}^{1,g,a}+\delta_{ART}^{1}X_{i,n,m,7,c,d}^{1,g,a}$$

$$K_{i,n,m,7,c,d}^{1,g,a}=\Gamma_{1,1}^{a}X_{i,n,m,6,c,d}^{1,g,a}-\delta_{ART}^{1}X_{i,n,m,7,c,d}^{1,g,a}$$

Where,

- $\Gamma_{n,m}^{a}$is the rate at which PWID initiate ART which varies by age $a$, NGO status $n$, and OAT status $m$
- $\delta_{ART}^{m}$ is the rate that PWID cease ART which varies by their OAT status $m$.
- We assume that ex-PWID are recruited onto ART at the same rate as PWID on OAT and in contact with NGOS (that is PWID with the highest recruitment rates).

## HCV infection $\mathbf{(C}_{\mathbf{i,n,m,h,c,d}}^{\mathbf{p,g,a}}\mathbf{)}$

$$C_{i,n,m,h,0,d}^{0,g,a}=-\beta_{i,n,m}^{g,a}X_{i,n,m,h,0,d}^{0,g,a}$$

$$C_{i,n,m,h,1,d}^{0,g,a}=\pi_{h}\beta_{i,n,m}^{g,a}X_{i,n,m,h,0,d}^{0,g,a}-{(1-\pi}_{h})\beta_{i,n,m}^{g,a}X_{i,n,m,h,1,d}^{0,g,a}$$

$$C_{i,n,m,h,2,d}^{0,g,a}={(1-\pi}_{h})\beta_{i,n,m}^{g,a}(X_{i,n,m,h,0,d}^{0,g,a}+X_{i,n,m,h,1,d}^{0,g,a})$$

$C_{i,n,m,h,c,d}^{1,g,0}=0$

Where,

- $\beta_{i,n,m,h}^{g,a}$ is the HCV force of infection through IDU
- $\pi_{h}$ is the proportion of HCV infections that spontaneously clear and depends on HIV status $h$.

## HCV force of infection through IDU $\boldsymbol{(\beta}_{\mathbf{i,n,m}}^{\mathbf{g,a}}\mathbf{)}$

For PWID in prison $(i=1)$

$$\beta_{1,n,m}^{g,a}=\lambda_{HCV}\hat{\eta}_{1,n,m}^{g}\frac{\sum_{g} \sum_{a} \sum_{n} \sum_{m} \sum_{h\neq6} \sum_{d} (T_{h}\hat{\eta}_{1,n,m}^{g}X_{i,n,m,h,2,d}^{0,g,a})}{\sum_{c} \sum_{g} \sum_{a} \sum_{n} \sum_{m} \sum_{h\neq6} \sum_{d} (\hat{\eta}_{1,n,m}^{g}X_{i,n,m,h,c,d}^{0,g,a})}$$

For PWID in the community $\left( i\neq1 \right)$ who are<25$(a=0)$

$$\beta_{i,n,m}^{g,0}={\lambda_{HCV}\hat{\eta}}_{i,n,m}^{g}\left[ \Psi\frac{\sum_{i\neq1} \sum_{g} \sum_{n} \sum_{m} \sum_{h\neq6} \sum_{d} \left( T_{h} \hat{\eta}_{i,n,m}^{g}X_{i,n,m,h,2,d}^{0,g,0} \right)}{\sum_{i\neq1} \sum_{c} \sum_{g} \sum_{n} \sum_{m} \sum_{h\neq6} \sum_{d} \left( \hat{\eta}_{i,n,m}^{g}X_{i,n,m,h,c,d}^{0,g,0} \right)}+\left( 1-\Psi\right)\frac{\sum_{i\neq1} \sum_{g} \sum_{a} \sum_{n} \sum_{m} \sum_{h\neq6} \sum_{d} \left( T_{h} \hat{\eta}_{i,n,m}^{g}X_{i,n,m,h,2,d}^{0,g,a} \right)}{\sum_{i\neq1} \sum_{c} \sum_{g} \sum_{a} \sum_{n} \sum_{m} \sum_{h\neq6} \sum_{d} \left( \hat{\eta}_{i,n,m}^{g}X_{i,n,m,h,c,d}^{0,g,a} \right)} \right]$$

For PWID in the community $\left( i\neq1 \right)$ who are $\geq$25$(a=1)$

$$\beta_{i,n,m}^{g,1}={\lambda_{HCV}\hat{\eta}}_{i,n,m}^{g}\left[ \Psi\frac{\sum_{i\neq1} \sum_{g} \sum_{n} \sum_{m} \sum_{h\neq6} \sum_{d} \left( T_{h} \hat{\eta}_{i,n,m}^{g}X_{i,n,m,h,2,d}^{0,g,1} \right)}{\sum_{i\neq1} \sum_{c} \sum_{g} \sum_{n} \sum_{m} \sum_{h\neq6} \sum_{d} \left( \hat{\eta}_{i,n,m}^{g}X_{i,n,m,h,c,d}^{0,g,1} \right)}+\left( 1-\Psi\right)\frac{\sum_{i\neq1} \sum_{g} \sum_{a} \sum_{n} \sum_{m} \sum_{h\neq6} \sum_{d} \left( T_{h} \hat{\eta}_{i,n,m}^{g}X_{i,n,m,h,2,d}^{0,g,a} \right)}{\sum_{i\neq1} \sum_{c} \sum_{g} \sum_{a} \sum_{n} \sum_{m} \sum_{h\neq6} \sum_{d} \left( \hat{\eta}_{i,n,m}^{g}X_{i,n,m,h,c,d}^{0,g,a} \right)} \right]$$

Where,

- $\lambda_{HCV}$ is the HCV transmission rate
- $\hat{\eta}_{i,n,m}^{g}$is the HCV injecting transmission risk of PWID relative to PWID who are male (g=0), have never been incarcerated (i=0), are not an NGO contact (n=0), and are not on OAT (m=0). By definition, $\hat{\eta}_{0,0,0}^{0}=1$
- HCV infectivity is heightened among HIV co-infected individuals(13). $T_{h}$ is the transmissibility of HCV which depends on HIV status h, and is defined as:

$$T_{h}=\left\{ \begin{aligned} 1 if h=0 \\ RR_{h} if h>0 \end{aligned} \right.$$

- ${RR}_{h}$ is the increase in HCV transmissibility if HIV positive.
- $\Psi$ is the amount of assortative mixing by age.

## HCV disease progression ($\mathbf{D}_{\mathbf{i,n,m,h,c,d}}^{\mathbf{p,g,a}}\mathbf{)}$

$D_{i,n,m,h,c,d}^{p,g,a}=0$ if c=0

$D_{i,n,m,h,c,d}^{p,g,a}=0$ if c=1 and d<4

$D_{i,n,m,h,2,0}^{p,g,a}=-\omega_{1}^{h}X_{i,n,m,h,2,0}^{p,g,a}$

$D_{i,n,m,h,2,1}^{p,g,a}=\omega_{1}^{h}X_{i,n,m,h,2,0}^{p,g,a}-\omega_{2}^{h}X_{i,n,m,h,2,1}^{p,g,a}$

$D_{i,n,m,h,2,2}^{p,g,a}=\omega_{2}^{h}X_{i,n,m,h,2,1}^{p,g,a}-\omega_{3}^{h}X_{i,n,m,h,2,2}^{p,g,a}$

$D_{i,n,m,h,2,3}^{p,g,a}=\omega_{3}^{h}X_{i,n,m,h,2,2}^{p,g,a}-\omega_{4}^{h}X_{i,n,m,h,2,3}^{p,g,a}$

$D_{i,n,m,h,2,4}^{p,g,a}=\omega_{4}^{h}X_{i,n,m,h,2,3}^{p,g,a}-{(\omega}_{D}^{2}+\omega_{H}^{2})X_{i,n,m,h,2,4}^{p,g,a}$

$$D_{i,n,m,h,1,4}^{p,g,a}=-{(\omega}_{D}^{1}+\omega_{H}^{1})X_{i,n,m,h,1,4}^{p,g,a}$$

$D_{i,n,m,h,c,5}^{p,g,a}=\omega_{D}^{c}X_{i,n,m,h,c,4}^{p,g,a}-(\mu_{D}^{h}+\omega_{H}^{c})X_{i,n,m,h,c,5}^{p,g,a}$

$D_{i,n,m,h,c,6}^{p,g,a}=\omega_{H}^{c}{(X}_{i,n,m,h,c,4}^{p,g,a}+X_{i,n,m,h,c,5}^{p,g,a})-\mu_{H}X_{i,n,m,h,c,6}^{p,g,a}$

Where,

- $\omega_{1}^{h}$ is the annual rate of progression from F0 to F1, which varies by HIV status $h.$
- $\omega_{2}^{h}$ is the annual rate of progression from F1 to F2, which varies by HIV status $h.$
- $\omega_{3}^{h}$ is the annual rate of progression from F2 to F3, which varies by HIV status $h.$
- $\omega_{4}^{h}$ is the annual rate of progression from F3 to F4, which varies by HIV status $h.$
- $\omega_{D}^{c}$ is the annual rate of progression from F4 to decompensated cirrhosis, which varies by HCV infection status $c$.
- $\omega_{H}^{c}$ is the annual rate of developing hepatocellular carcinoma from F4 or decompensated cirrhosis, which varies by HCV infection status $c.$
- $\mu_{D}^{h}$ is the annual rate of mortality from decompensated cirrhosis, which depends on HIV status $h.$
- $\mu_{H}$ is the annual rate of mortality from hepatocellular carcinoma.

# Model Parameterisation

The model is primarily parameterised using data from the 2011 (n=9,069), 2013 (n=9,502), 2015 (n=9,405) and 2017 (n=10,076) national IBBA surveys(3-6) and the 2015 Expanding Medication-Assisted Therapy (ExMAT) bio-behavioural survey (n=1,612)(7). The IBBAs, conducted by the Alliance for Public Health (APH), recruited PWID using respondent driven sampling (RDS) from 26-30 cities per round. The ExMAT survey recruited PWID using stratified sampling in five cities, with random sampling of PWID currently or ever on OAT and an RDS primarily recruiting PWID never on OAT. The Centre for Public Health‘s (CPH) national HIV treatment database was used to estimate annual rates of ART LTFU among PWID. Additionally, follow-up data from the standard-of-care arm of a recent randomised controlled trial in Kyiv(8) gave mortality rates among PWID.

Supplementary Table 1: Parameter table.

| **Parameter** | | **Symbol** | **Prior Distribution*** | **Posterior range** | **Source/Notes** |
| --- | --- | --- | --- | --- | --- |
|  | **Demographic Parameters** | | | | |
| Average duration of injecting (years) | | ${\frac{1}{\mu}}_{c}$ | Uniform distribution with range 7.5 – 50 | 13.86-36.41 | 2011/13/15/17 APH IBBAs |
| Non-disease related death rate among PWID (per 100py) | | $\mu_{0}$ | Lognormal distribution with mean 3.99 and 95%CI: 1.99 - 7.14 | 5.77-7.13 | (8) |
| Proportion of injecting initiates that are male | | $\sum_{i,a} p_{i}^{0,a}$ | Uniform distribution with range 0.7 – 0.85 | 0.75-0.80 | Calibrated |
| Proportion of injecting initiates that are aged <25 years old | | $\sum_{i,g} p_{i}^{g,o}$ | Uniform distribution with range 0.66-0.86 | 0.69-0.81 | 2011/13/15/17 APH IBBAs |
| Average age at which PWID start injecting if <25 when start | | $\zeta_{0}$ | Uniform distribution with range 16 – 20 | 19-20 | 2011/13/15/17 APH IBBAs |
| Factor reduction in the number of people initiating IDU | | $\Delta_{\mathrm{change}}$ | Uniform distribution with range 0 – 1 | 0.30-0.63 | Calibrated |
| Year in which initiation of IDU reduces | | $yr_{\mathrm{change}}$ | Uniform distribution with range 1990 – 2000 | 1990.1-1993.4 | Based on PWID population size estimates. |
| Length of time over which the number of new PWID initiates per year decreases | | $d_{\mathrm{change}}$ | Uniform distribution with range 0 - 10 years | 0.78-4.26 | Calibrated |
|  | | $N_{0}$ | Uniform distribution with range 450,000-1,000,000 | 565,532-794,407 | Calibrated |
| HIV seed prevalence in 1985 | | N/A | Uniform distribution with range 0.01 – 0.1 | 0.03-0.05 | Calibrated |
|  | **Incarceration Parameters** | | | | |
| Average length of each incarceration episode (months) | | $\frac{12}{\tau}$ | Uniform distribution with range 13 – 15 | 14.1-15.0 | 2011/13/15/17 APH IBBAs;  Exmat |
| Proportion of females that have never been incarcerated when initiating IDU | | $p_{0}^{1,a}$ | Uniform distribution with range 0.95 – 1.0 | 0.97-1.0 | Exmat |
| Proportion of males that have never been incarcerated when initiating IDU | | $p_{0}^{0,a}$ | Uniform distribution with range 0.82 – 1.0 | 0.83-0.91 | Exmat |
| Proportion of individuals who initiate IDU with a history of incarceration that first inject in prison | | $\frac{p_{1}^{g,a}}{p_{1}^{g,a}+p_{2}^{g,a}}$ | Uniform distribution with range 0.0 – 1.0 | 0.67-0.99 | Calibrated |
| Primary incarceration rate amongst young female PWID | | $\gamma_{0,0}^{1,0}$ | Uniform distribution with range 0.0 – 0.3 | 0.00-0.017 | Calibrated |
| Primary incarceration rate amongst young male PWID | | $\gamma_{0,0}^{0,0}$ | Uniform distribution with range 0.0 – 0.3 | 0.00-0.033 | Calibrated |
| Primary incarceration rate amongst old female PWID | | $\gamma_{0,0}^{1,1}$ | Uniform distribution with range 0.0 – 0.3 | 0.027-0.063 | Calibrated |
| Primary incarceration rate amongst old male PWID | | $\gamma_{0,0}^{0,1}$ | Uniform distribution with range 0.0 – 0.3 | 0.049-0.083 | Calibrated |
| Re-incarceration rate amongst young female PWID | | $\gamma_{1,0}^{1,0}$ | Uniform distribution with range 0.0 – 1.0 | 0.57-0.96 | Calibrated |
| Re-incarceration rate amongst young male PWID | | $\gamma_{1,0}^{0,0}$ | Uniform distribution with range 0.0 – 1.0 | 0.240.48 | Calibrated |
| Re-incarceration rate amongst old female PWID | | $\gamma_{1,0}^{0,1}$ | Uniform distribution with range 0.0 – 1.0 | 00.00-0.06 | Calibrated |
| Re-incarceration rate amongst old male PWID | | $\gamma_{1,0}^{1,1}$ | Uniform distribution with range 0.0 – 1.0 | 0.017-0.12 | Calibrated |
|  | **HIV Natural History Parameters** | | | | |
| Average duration of acute stage of HIV infection (Months) | | $\frac{12}{h_{a}}$ | Triangular distribution with peak at 2.9 and range 1.23 – 6.00 | 3.24-5.32 | (9) |
| Average duration of pre-AIDS stage of HIV infection (Months) | | $\frac{12}{h_{p}}$ | Triangular distribution with peak at 9.0 and range 4.81 – 14.0 | 5.86-10.52 | (9) |
| Average duration from infection to AIDS (Years) | | $\frac{1}{h_{c}}+\frac{1}{h_{a}}+\frac{1}{h_{p}}$ | Uniform distribution with range 10.8 – 16.7 | 10.80-13.39 | (10) |
| Average time until death from AIDS if not on ART (Months) | | $\frac{12}{\mu_{A}}$ | Lognormal distribution with mean 10 and 95%CI: 6.79 – 12.7 | 8.64-12.33 | (9) |
| HIV transmissibility if in acute stage of infection | | $\alpha_{1}*\alpha_{2}$ | Lognormal distribution with mean 276 and 95%CI: 131 – 509 | 208.18-336.31 | (9) |
| HIV transmissibility if in latent stage of infection | | $\alpha_{2}$ | Lognormal distribution with mean 10.6 and 95%CI: 7.61 – 13.3 | 9.44-13.01 | (9) |
| HIV transmissibility if in pre-AIDS stage of infection | | $\alpha_{3}*\alpha_{2}$ | Lognormal distribution with mean 76.0 and 95%CI: 41.3 – 128.0 | 41.4-82.09 | (9) |
|  | **ART Parameters** | | | | |
| ART recruitment rate per year if >=25 years old and not on OAT or NGO contact | | $\Gamma_{0,0}^{1}$ | Uniform distribution with range 0.0 – 0.3 | 0.050-0.12 | Calibrated |
| Relative ART recruitment rate if <25 years old vs >=25 years old | | $\frac{\Gamma_{0,0}^{0}}{\Gamma_{0,0}^{1}}$ | Uniform distribution with range 0.0 – 1.0 | 0.33-0.70 | Calibrated |
| Rate of loss to care from ART (per 100 py) if not on OAT | | $\delta_{ART}^{0}$ | Uniform distribution with range 10.9– 15.8 | 12.53-15.10 | CPH HIV treatment database |
| Proportion of PWID on ART who are virally supressed if not on OAT | | $V_{0}$ | Lognormal distribution with mean 69% and 95%CI: 49-77% | 49.00-59.63 | (11) |
| Average log viral load if not on ART | | $v_{b}$ | Triangular distribution with peak at 4.79 and range 4.11 – 5.27 | 4.50-5.03 | (12-14) |
| Average reduction in log viral load if on ART and not virally suppressed | | $\Delta_{u}$ | Triangular distribution with peak at 0.81 and range 0.0 – 2.27 | 0.11-0.88 | (12-14) |
| Factor difference in HIV transmission risk for each log increment PVL | | $r_{t}$ | Lognormal distribution with mean 2.45 and 95%CI: 1.85 – 3.26 | 2.53-3.26 | (1) |
| Factor reduction in HIV disease progression/mortality if on ART | | $A$ | Lognormal distribution with mean 0.34 and 95%CI: 0.26-0.44 | 0.26-0.35 | (15) |
|  | **HCV Natural History Parameters** | | | | |
| Proportion of infections that spontaneous clear if HIV negative | | $\pi_{0}$ | Normal distribution with mean 0.254 and 95%CI: 0.218 – 0.292 | 0.22-0.26 | (16) |
| Odds ratio of clearing HCV infection if HIV positive vs. HIV negative | | $\frac{\pi_{1}(1-\pi_{0})}{\left( 1-\pi_{1} \right)\pi_{0}}$ | Lognormal distribution with mean 0.58 and 95%CI: 0.38 – 0.88 | 0.53-0.81 | (16) |
| Increase in HCV infectivity if HIV co-infected | | $RR_{h}$ | Uniform distribution with range 1.0 – 7.0 | 1.01-3.03 | (17) |
| Annual rate of progression from F0 to F1 in HIV negatives with chronic HCV infection | | $\omega_{1}^{0}$ | Normal distribution with mean 0.128 and  95%CI: 0.08 – 0.176 | 0.08-0.176 | (18) |
| Annual rate of progression from F1 to F2 in HIV negatives with chronic HCV infection | | $\omega_{2}^{0}$ | Normal distribution with mean 0.059 and  95%CI: 0.035 – 0.082 | 0.035-0.082 | (18) |
| Annual rate of progression from F2 to F3 in HIV negatives with chronic HCV infection | | $\omega_{3}^{0}$ | Normal distribution with mean 0.079 and  95%CI: 0.056 – 0.10 | 0.056-0.10 | (18) |
| Annual rate of progression from F3 to F4 in HIV negatives with chronic HCV infection | | $\omega_{4}^{0}$ | Normal distribution with mean 0.116 and  95%CI: 0.07 – 0.161 | 0.07-0.161 | (18) |
| Relative risk of progression to cirrhosis if on ART vs HIV positive not on ART | | $\frac{\omega_{d}^{3}}{\omega_{d}^{2}}$ | Uniform distribution with range 0.27 – 0.70 | 0.27-0.70 | (19, 20) |
| Relative risk of progression to cirrhosis if HIV positive | | $\frac{\omega_{d}^{2}}{\omega_{d}^{0}}$ | Lognormal distribution with mean 2.489 and 95%CI: 1.811 – 3.42 | 1.811-3.42 | (19) |
| Annual probability of progression to decompensated cirrhosis from F4 in individuals with chronic HCV infection | | $1-e^{-\omega_{D}^{2}}$ | Beta distribution with shape parameters 14.6168 and 360.1732 | 0.014-0.081 | (21) |
| Annual probability of death from decompensated cirrhosis if HIV negative | | $1-e^{-\mu_{D}^{0}}$ | Beta distribution with shape parameters 147.03 and 983.97 | 0.099-0.17 | (21) |
| Relative risk of mortality from decompensated cirrhosis if HIV positive vs negative | | $\frac{\mu_{D}^{h}}{\mu_{D}^{0}}$  $(h>0)$ | Lognormal distribution with mean 2.26 and 95%CI: 1.51 – 3.38 | 1.51-3.38 | (22, 23) |
| Annual probability of progression to HCC from F4 or decompensated cirrhosis in individuals with chronic HCV infection. | | $1-e^{-\omega_{H}^{c}}$ | Beta distribution with shape parameters 1.9326 and 136.1732 | 0.00014-0.067 | (21) |
| Relative risk for progression rate from compensated cirrhosis to HCC following SVR | | $\frac{\omega_{H}^{1}}{\omega_{H}^{2}}$ | Lognormal distribution with mean 0.23 and 95%CI: 0.16 – 0.35 | 0.16-0.35 | (24) |
| Annual probability of death from HCC | | $1-e^{-\mu_{h}}$ | Beta distribution with shape parameters 117.1033 and 155.23 | 0.34-0.55 | (21) |
|  | **HIV/HCV injecting transmission parameters** | | | | |
| Relative injecting risk if female vs male | | $\frac{\hat{\eta}_{i,n,m}^{1,a}}{\hat{\eta}_{i,n,m}^{0,a}}=\frac{\eta_{i,n,m}^{1,a}}{\eta_{i,n,m}^{0,a}}$ | Lognormal distribution with mean 1.91 and 95%CI: 1.75 – 2.10 | 1.42-1.71 | 2011/13/15/17 APH IBBAs - analyses of frequency injected with used equipment in the last month |
| Relative injecting risk if an NGO contact vs not | | $\frac{\hat{\eta}_{i,n,m}^{g,a}}{\hat{\eta}_{i,n,m}^{g,a}}=\frac{\eta_{i,n,m}^{g,a}}{\eta_{i,n,m}^{g,a}}$ | Lognormal distribution with mean 0.73 and 95%CI: 0.55 – 0.97 | 0.63-0.86 |  |
| Relative injecting risk if recently incarcerated (last 12 months) vs never incarcerated | | $\frac{\hat{\eta}_{i,n,m}^{g,a}}{\hat{\eta}_{i,0,m}^{g,a}}=\frac{\eta_{i,n,m}^{g,a}}{\eta_{i,0,m}^{g,a}}$  $(n=1,2)$ | Lognormal distribution with mean 2.07 and 95%CI: 1.35-3.20 | 1.35-2.04 |  |
| Relative injecting risk if ever incarcerated (not in last 12 months) vs never incarcerated | | $\frac{\hat{\eta}_{2,n,m}^{g,a}}{\hat{\eta}_{0,n,m}^{g,a}}=\frac{\eta_{2,n,m}^{g,a}}{\eta_{0,n,m}^{g,a}}$ | Lognormal distribution with mean 1.69 and 95%CI: 1.27 – 2.25 | 1.27-1.70 |  |
| Relative injecting risk if currently incarcerated vs in injecting risk if in prison vs never incarcerated | | $\frac{\hat{\eta}_{1,n,m}^{g,a}}{\hat{\eta}_{0,n,m}^{g,a}}=\frac{\eta_{1,n,m}^{g,a}}{\eta_{0,n,m}^{g,a}}$ | Uniform distribution with range 0.0 – 10.0 | 0.00-0.83 | Calibrated |
| HIV transmission rate through IDU | | $\lambda_{HIV}$ | Uniform distribution with range 0.0 – 0.3 | 0.053-0.079 | Calibrated |
| HCV transmission rate through IDU | | $\lambda_{HCV}$ | Uniform distribution with range 0.0 – 0.5 | 0.14-0.26 | Calibrated |
| % of mixing that is assortative by age. | | $\Psi$ | Uniform distribution with range 0.0-0.5 | 0.00-0.27 | Calibrated. RDS recruitment data from IBBAs suggest assortative recruitment by age |
|  | **HIV Sexual Transmission Parameters** | | | | |
| Efficacy of condoms in reducing HIV transmission risk | | $\zeta_{c}$ | Triangular distribution with peak at 0.8 and range 0.66 – 0.94 | 0.78-0.93 | (25) |
| Baseline odds of using a condom | | $\frac{C^{0,0,0,0,0}}{1-C^{0,0,0,0,0}}$ | Lognormal distribution with mean 1.59 and 95%CI: 1.37 – 1.84 | 1.39-1.64 | 2011/13/15/17 APH IBBAs |
| OR of using a condom if ever incarcerated (recent or non-recent) vs never incarcerated | | $\frac{(C^{g,a,i,n,m})(1-C^{g,a,0,n,m})}{(1-C^{g,a,i,n,m})(C^{g,a,0,n,m})}$  $(i=2,3)$ | Lognormal distribution with mean 0.70 and 95%CI: 0.65 – 0.75 | 0.65-0.70 | 2011/13/15/17 APH IBBAs |
| OR of using a condom if on OAT vs not on OAT | | $\frac{(C^{g,a,i,n,m})(1-C^{g,a,i,n,0})}{(1-C^{g,a,i,n,m})(C^{g,a,i,n,0})}$  $(m=1,2)$ | Lognormal distribution with mean 1.27 and 95%CI: 1.08 – 1.48 | 1.10-1.26 | 2011/13/15/17 APH IBBAs |
| OR of using a condom if Female vs male | | $\frac{(C^{1,a,i,n,m})(1-C^{0,a,i,n,m})}{(1-C^{1,a,i,n,m})(C^{0,a,i,n,m})}$ | Lognormal distribution with mean 0.58 and 95%CI: 0.54 – 0.63 | 0.58-0.63 | 2011/13/15/17 APH IBBAs |
| OR of using a condom if NGO contact vs not | | $\frac{\left( C^{g,a,i,n,m} \right)\left( 1-C^{g,a,i,0,m} \right)}{\left( 1-C^{g,a,i,n,m} \right)\left( C^{g,a,i,0,m} \right)}$  $(n=1,2)$ | Lognormal distribution with mean 1.33 and 95%CI: 1.24 – 1.44 | 1.25-1.35 | 2011/13/15/17 APH IBBAs |
| OR of using a condom if old (>=25) vs young (<=25) | | $\frac{(C^{g,1,i,n,m})(1-C^{g,0,i,n,m})}{(1-C^{g,1,i,n,m})(C^{g,0,i,n,m})}$ | Lognormal distribution with mean 0.65 and 95%CI: 0.57 – 0.72 | 0.56-0.63 | 2011/13/15/17 APH IBBAs |
| Baseline odds of being sexually active | | $\frac{m_{0,0,0}^{0,0}}{1-m_{0,0,0}^{0,0}}$ | Lognormal distribution with mean 8.16 and 95%CI: 6.45 – 10.33 | 6.79-8.34 | 2011/13/15/17 APH IBBAs |
| OR of being sexually active if old (>=25) vs young (<=25) | | $\frac{m_{i,n,m}^{g,1}(1-m_{i,n,m}^{g,0})}{{(1-m_{i,n,m}^{g,1})m}_{i,n,m}^{g,0}}$ | Lognormal distribution with mean 0.71 and 95%CI: 0.60 – 0.83 | 0.70-0.83 | 2011/13/15/17 APH IBBAs |
| OR of being sexually active if on OAT vs not on OAT | | $\frac{m_{i,n,m'}^{g,a}(1-m_{i,n,0}^{g,a})}{{(1-m_{i,n,m'}^{g,a})m}_{i,n,0}^{g,a}}$  $(m^{'}=1,2)$ | Lognormal distribution with mean 0.74 and 95%CI: 0.62 – 0.88 | 0.62-0.71 | 2011/13/15/17 APH IBBAs |
| OR of being sexually active if ever incarcerated but not recently vs never incarcerated | | $\frac{m_{3,n,m}^{g,a}(1-m_{0,n,m}^{g,a})}{{(1-m_{3,n,m}^{g,a})m}_{0,n,m}^{g,a}}$ | Lognormal distribution with mean 0.91 and 95%CI: 0.83 – 0.99 | 0.84-0.92 | 2011/13/15/17 APH IBBAs |
| OR of being sexually active if recently incarcerated vs never incarcerated | | $\frac{m_{2,n,m}^{g,a}(1-m_{0,n,m}^{g,a})}{{(1-m_{2,n,m}^{g,a})m}_{0,n,m}^{g,a}}$ | Lognormal distribution with mean 0.79 and 95%CI: 0.69 – 0.91 | 0.72-0.82 | 2011/13/15/17 APH IBBAs |
| OR of being sexually active if female vs male | | $\frac{m_{i,n,m}^{1,a}(1-m_{i,n,m}^{0,a})}{{(1-m_{i,n,m}^{1,a})m}_{i,n,m}^{0,a}}$ | Lognormal distribution with mean 1.18 and 95%CI: 1.06 – 1.30 | 1.07-1.16 | 2011/13/15/17 APH IBBAs |
| Baseline annual number of sexual contacts | | $s_{0,0,0}^{0,0}$ | Lognormal distribution with mean 117.40 and 95%CI: 106.68 – 129.19 | 108.17-119.88 | 2011/13/15/17 APH IBBAs |
| Relative difference in number of sexual contacts among those sexually active if old (>=25) vs young (<=25) | | $\frac{s_{i,n,m}^{g,1}}{s_{i,n,m}^{g,0}}$ | Lognormal distribution with mean 0.82 and 95%CI: 0.78 – 0.87 | 0.78-0.82 | 2011/13/15/17 APH IBBAs |
| Relative difference in number of sexual contacts among those sexually active if on OST vs not on OST | | $\frac{s_{i,n,m}^{g,a}}{s_{i,n,0}^{g,a}}$  $(m=1,2)$ | Lognormal distribution with mean 0.86 and 95%CI: 0.80 – 0.92 | 0.80-0.88 | 2011/13/15/17 APH IBBAs |
| Relative difference in number of sexual contacts among those sexually active if ever incarcerated but not in last 12 months vs never incarcerated | | $\frac{s_{3,n,m}^{g,a}}{s_{0,n,m}^{g,a}}$ | Lognormal distribution with mean 1.05 and 95%CI: 1.01 - 1.08 | 1.04-1.08 | 2011/13/15/17 APH IBBAs |
| Relative difference in number of sexual contacts among those sexually active if recently incarcerated in last 12 months vs never incarcerated | | $\frac{s_{2,n,m}^{g,a}}{s_{0,n,m}^{g,a}}$ | Lognormal distribution with mean 1.19 and 95%CI: 1.13 – 1.26 | 1.15-1.24 | 2011/13/15/17 APH IBBAs |
| HIV sexual transmission rate for males | | $\lambda^{0}$ | Uniform distribution with range 0.0 – 0.3 | 0.00035-0.00096 | Calibrated |
| Relative increase in HIV sexual transmission rate for females | | $\frac{\lambda^{1}}{\lambda^{0}}$ | Uniform distribution with range 1.0 – 3.0 | 1.00-1.86 | (26) |
| Proportion of male PWID's partners that are PWID | | $L^{0}$ | Triangular distribution with peak at 0.127 and range 0.114 – 0.140 | 0.114-0.127 | 2011/13/15/17 APH IBBAs |
|  | **NGO Parameters** | | | | |
| Rate of starting contact with NGO in 2011 if HIV negative and >25 years old | | $\kappa_{0}^{0}$ | Uniform distribution with range 0.0 – 1.0 | 0.10-0.28 | Calibrated; NGO recruitment rate increases linearly from 0 in 1997 to  $\kappa_{0}^{0}$ in 2011 |
| Relative risk of starting contact with NGO if HIV positive vs HIV negative | | $\frac{\kappa_{0}^{a}}{\kappa_{h}^{a}}$  $(h>0)$ | Uniform distribution with range 1.0 – 5.0 | 1.00-2.43 | Calibrated |
| Relative risk of starting contact with NGO if young (<=25) vs. old (>25) | | $\frac{\kappa_{h}^{0}}{\kappa_{h}^{1}}$ | Uniform distribution with range 0.0 – 1.0 | 0.66-0.97 | Calibrated |
| Rate of ceasing NGO contact | | $\delta_{NGO}$ | Uniform distribution with range 0.0 – 2.0 | 0.045-0.15 | Calibrated |
| RR of starting ART recruitment if in contact with NGO | | $\frac{\Gamma_{n,m}}{\Gamma_{0,m}}$  $(n=1,2)$ | Uniform distribution with range 1.0 – 5.0 | 2.59-4.18 | Calibrated |
| RR of starting OAT if not in contact with NGO | | $\frac{l_{0}}{l_{n}}$  $(n=1,2)$ | Uniform distribution with range 0.0-1.0 | 0.00-0.11 | Calibrated |
|  | **OAT Parameters** | | | | |
| OAT recruitment rate if not in contact with NGOs. | | $l_{0}$ | Uniform distribution with range 0.0 – 0.02 | 0.026-0.055 | Calibrated |
| Rate of loss to care from OAT if on OAT for <2 years | | $\delta_{OAT}^{1}$ | Uniform distribution with range 0.45 – 0.50 | 0.46-0.50 | Estimated using data from (27) |
| Rate of loss to care from OAT if on OAT for >=2 years | | $\delta_{OAT}^{2}$ | Uniform distribution with range 0.1 – 0.15 | 0.10-0.13 |  |
| RR of starting ART if on OAT vs not on OAT | | $\frac{\Gamma_{n,m}}{\Gamma_{n,m^{'}}}$  $(m=1,2; m’ = 0,3)$ | Lognormal distribution with mean 1.87 and 95%CI: 1.50 – 2.33 | 1.78-2.27 | (28) |
| OR of being virally supressed among those on ART if on OAT vs not on OAT | | $\frac{V_{m}(1-V_{m'})}{{(1-V_{m})V}_{m^{'}}}$  $(m=1,2; m’ = 0,3)$ | Lognormal distribution with mean 1.45 and 95%CI: 1.21 – 1.73. | 1.24-1.58 | (28) |
| RR of ART loss to care if on OAT vs not on OAT | | $\frac{\delta_{ART}^{m}}{\delta_{ART}^{m^{'}}}$  $(m=1,2; m’ = 0,3)$ | Lognormal distribution with mean 0.77 and 95%CI: 0.63 – 0.95 | 0.73-0.94 | (28) |
| RR of HCV transmission through IDU if on OAT vs not on OAT | | $\frac{\hat{\eta}_{i,n,m}^{g,a}}{\hat{\eta}_{i,n,m'}^{g,a}}$  $(m=1,2; m’ = 0,3)$ | Lognormal distribution with mean 0.46 and 95%CI: 0.32 – 0.67 | 0.43-0.55 | (29) |
| RR of HIV transmission through IDU if on OAT vs not on OAT | | $\frac{\eta_{i,n,m}^{g,a}}{\eta_{i,n,m'}^{g,a}}$  $(m=1,2; m’ = 0,3)$ | Lognormal distribution with mean 0.50 and 95%CI: 0.40 – 0.63 | 0.52-0.67 | (30) |
| RR of incarceration if on OAT vs not on OAT | | $\frac{\gamma_{0,m}^{g,a}}{\gamma_{0,m'}^{g,a}}=\frac{\gamma_{1,m}^{g,a}}{\gamma_{1,m'}^{g,a}}$  $(m=1,2; m’ = 0,3)$ | Uniform distribution with range 0.58 – 0.90 | 0.78-0.90 | (31, 32) |
| RR of non-disease related mortality if on OAT vs not on OAT | | $RR_{\mathrm{OAT}}^{\mu_{0}}$ | Lognormal distribution with mean 0.33 and 95%CI: 0.28 – 0.39 | 0.28-0.32 | (33) |
| RR of non-disease related mortality in first 4 weeks after starting OAT vs rest of time on OAT | | $\epsilon_{0}$ | Lognormal distribution with mean 1.97 and 95%CI: 0.94 – 4.10 | 1.86-3.50 | (34) |
| RR of non-disease related mortality in first 4 weeks after leaving OAT | | $\epsilon_{1}$ | Lognormal distribution with mean 2.38 and 95%CI: 1.51 – 3.74 | 2.81-3.66 | (34) |
| * Uniform priors were used for ‘uninformative priors’ whereby only calibration data was available to inform these parameters. Triangular distributions were used when there were empirical estimates but their uncertainty measures (e.g. IQR, or range across multiple estimates) did not merit lognormal/normal distributions. | | | | | |

# Model Calibration

A simplified model without ex-PWID and HCV disease progression is used for model calibration, assuming that HCV deaths will not occur in the time frame of a PWID’s duration of injecting (prior: 7.5-50 years). After model calibration, for each model run we sample parameters related to HCV disease progression and mortality. Model projections with HCV disease progression agreed with those obtained during model calibration.

The model was calibrated using an approximate Bayesian computation Sequential Monte Carlo (ABC SMC) method(35) to data on the: PWID population size; proportion of PWID that are female; proportion of PWID that are young; HIV and HCV antibody prevalences and differences in prevalence by age, gender and incarceration status (never vs ever incarcerated); difference in HCV antibody prevalence between HIV-positive PWID and HIV-negative PWID; proportion of PWID who have ever been incarcerated, or incarcerated in the last 12 months; OAT coverage and difference by NGO status; ART coverage and differences by age and NGO status; coverage of NGOs and differences by age and HIV-status; and proportion that have been contacts for <2 years. Supplementary table 2 shows the calibration data used in the SMC routine.

The ABC SMC(35) is an iterative algorithm that begins by sampling 1,000 parameter sets from the prior distributions and evaluating for each parameter set the goodness of fit. The goodness of fit (G) for a parameter set was defined as:

$$G=\sum_{j} {|ln(m}_{j})-{ln(d}_{j})|*\left( 1-I\left( m_{j},l_{j},u_{j} \right) \right)$$

where,

- $m_{j}$ is the model projection for summary statistic $j.$
- $d_{j}$ is the data estimate for summary statistic $j.$
- $l_{j}$ is lower bound of the 95% confidence interval for summary statistic $j.$
- $u_{j}$ is upper bound of the 95% confidence interval for summary statistic $j.$
- $I\left( m_{j},l_{j},u_{j} \right)$ is the function defined as:

$$I\left( m_{j},l_{j},u_{j} \right)=\left\{ \begin{aligned} 1 if l_{j}\leq m_{j}\leq u_{j,} \\ 0 otherwise \end{aligned} \right.$$

The tolerance of the next iteration is then set as the 80th percentile of the goodness of fits of the 1,000 sampled parameter sets and the next iteration begins. At subsequent iterations, parameter sets from the previous iteration are sampled from, with weights dependent upon the prior likelihood of the parameter set and the perturbation kernel. These sampled parameter sets are then perturbed using a uniform perturbation kernel, which could perturbate each parameter by at most +/- 5% of the prior range, so as to still be within the prior ranges for each parameter, accepting those that gave model fits whose goodness of fit, measured by the distance function, was better than the 80^th^ percentile of accepted model fits in the previous iteration (the tolerance). Sampling of parameter sets continues, until 1,000 are accepted. The tolerance of the next iteration is then set as the 80th percentile of the goodness of fits of the accepted parameter sets and the next iteration begins. The ABC SMC routine continued iteratively until successive iterations no longer improve the goodness of fit.

**Supplementary Table 2: Data used in model calibration**

|  | **Value (95%CI)** | **Date of Value** | **Source** |
| --- | --- | --- | --- |
| **Demographics** | | | |
| PWID Population Size | 560000 (448000 - 672000) | 2002 | (36) |
|  | 380000 (325000 - 425000) | 2005 | (36) |
|  | 295000 (230000 - 360000) | 2009 | (36) |
|  | 310000 (274000 - 388000) | 2011 | (36) |
|  | 355000 (290000 - 440000) | 2013 | (36) |
|  | 358700 (265200 - 475700) | 2015 | (37) |
|  | 356500 (255500 - 475000) | 2018 | (38) |
| Proportion of PWID that are female | 24.4% (22.6 - 25.7) | 2009 | 2009 APH IBBA |
|  | 26.3% (25.3 - 27.1) | 2011 | 2011 APH IBBA |
|  | 23.2% (21.7 - 23.8) | 2013 | 2013 APH IBBA |
|  | 20.1% (18.6 - 20.8) | 2015 | 2015 APH IBBA |
|  | 18.1% (17.5 - 18.5) | 2017 | 2017 APH IBBA |
| Proportion of PWID that are young | 10.0% (Uncertainty Interval: 0.0-20.0) | 2015 | ~10% of PWID are aged <25 across IBBA surveys. We incorporate wide uncertainty to account for likely under sampling of young PWID. |
| **Incarceration History** | | | |
| Proportion of males <=25 that have ever been incarcerated | 8.4% (5.7 - 10.7) | 2011 | 2011 APH IBBA |
|  | 6.5% (3.7 - 10.6) | 2013 | 2013 APH IBBA |
|  | 11.9% (8.8 - 13.9) | 2015 | 2015 APH IBBA |
|  | 8.6% (5.6 - 10.6) | 2017 | 2017 APH IBBA |
| Proportion of females <=25 that have ever been incarcerated | 8.1% (0.7 - 16.2) | 2011 | 2011 APH IBBA |
|  | 3.7% (0.8 - 7.4) | 2013 | 2013 APH IBBA |
|  | 1.2% (0.4 - 2.3) | 2015 | 2015 APH IBBA |
|  | 3.6% (1.0 - 6.3) | 2017 | 2017 APH IBBA |
| Proportion of males >25 that have ever been incarcerated | 42.2% (40.7 - 43.1) | 2011 | 2011 APH IBBA |
|  | 40.5% (39.8 - 42.4) | 2013 | 2013 APH IBBA |
|  | 44.6% (43.5 - 46.2) | 2015 | 2015 APH IBBA |
|  | 46.0% (44.6 - 47.7) | 2017 | 2017 APH IBBA |
| Proportion of females >25 that have ever been incarcerated | 25.1% (23 - 27.6) | 2011 | 2011 APH IBBA |
|  | 33.2% (31.5 - 36.6) | 2013 | 2013 APH IBBA |
|  | 24.5% (22 - 27.8) | 2015 | 2015 APH IBBA |
|  | 31.6% (28.6 - 38.8) | 2017 | 2017 APH IBBA |
| Proportion of males <=25 that have been incarcerated in the last year | 3.8% (3.0 - 4.4) | 2011 | 2011 APH IBBA |
|  | 3.5% (2.5 - 4.9) | 2013 | 2013 APH IBBA |
|  | 5.2% (3.1 - 6.4) | 2017 | 2017 APH IBBA |
| Proportion of females <=25 that have been incarcerated in the last year | 1.0% (0.1 - 1.9) | 2011 | 2011 APH IBBA |
|  | 1.7% (0.3 - 3.1) | 2013 | 2013 APH IBBA |
|  | 1.5% (0.0 - 3.3) | 2017 | 2017 APH IBBA |
| Proportion of males >25 that have been incarcerated in the last year | 5.8% (5.0 - 6.5) | 2011 | 2011 APH IBBA |
|  | 5.9% (5.4 - 6.2) | 2013 | 2013 APH IBBA |
|  | 5.8% (5.1 - 6.3) | 2017 | 2017 APH IBBA |
| Proportion of females >25 that have been incarcerated in the last year | 2.2% (1.4 - 4.3) | 2011 | 2011 APH IBBA |
|  | 2.4% (1.0 - 4.3) | 2013 | 2013 APH IBBA |
|  | 2.4% (0.8 - 3.5) | 2017 | 2017 APH IBBA |
| **HIV Prevalence** | | | |
| HIV prevalence among male community PWID aged >25 | 22.6% (20.7-23.6) | 2011 | 2011 APH IBBA |
|  | 18.3% (17.3-19.6) | 2013 | 2013 APH IBBA |
|  | 20.8% (19.3.-21.5) | 2015 | 2015 APH IBBA |
|  | 20.9% (20.5-21.7) | 2017 | 2017 APH IBBA |
| OR of being HIV positive if ever incarcerated vs never incarcerated |  |  |  |
|  | 2.66 (2.39 - 2.96) | 2011 | 2011 APH IBBA |
|  | 2.64 (2.36 - 2.95) | 2013 | 2013 APH IBBA |
|  | 2.46 (2.21 - 2.72) | 2015 | 2015 APH IBBA |
|  | 2.23 (2.02 - 2.47) | 2017 | 2017 APH IBBA |
| OR of being HIV positive if female vs male |  |  |  |
|  | 1.19 (1.06 - 1.34) | 2011 | 2011 APH IBBA |
|  | 1.48 (1.31 - 1.67) | 2013 | 2013 APH IBBA |
|  | 1.45 (1.28 - 1.63) | 2015 | 2015 APH IBBA |
|  | 1.83 (1.63 - 2.06) | 2017 | 2017 APH IBBA |
| OR of being HIV positive if <=25 vs >25 | 0.29 (0.23 - 0.37) | 2009 | 2009 APH IBBA |
|  | 0.27 (0.22 - 0.32) | 2011 | 2011 APH IBBA |
|  | 0.19 (0.15 - 0.26) | 2013 | 2013 APH IBBA |
|  | 0.15 (0.11 - 0.21) | 2015 | 2015 APH IBBA |
|  | 0.13 (0.09 - 0.2) | 2017 | 2017 APH IBBA |
| **HCV Prevalence (Antibody)** | | | |
| HCV prevalence among male community PWID aged >25 | 56.2% (54.3-59.1) | 2013 | 2013 APH IBBA |
|  | 55.2% (54.6-58.7) | 2015 | 2015 APH IBBA |
|  | 65.8% (65.2-67.6) | 2017 | 2017 APH IBBA |
| OR of being HCV positive if HIV positive vs HIV negative | 4.94 (4.30 - 5.67) | 2013 | 2013 APH IBBA |
| OR of being HCV positive if HIV positive vs HIV negative | 3.09 (2.75 - 3.46) | 2015 | 2015 APH IBBA |
| OR of being HCV positive if HIV positive vs HIV negative | 3.32 (2.93 - 3.77) | 2017 | 2017 APH IBBA |
| OR of being HCV positive if ever incarcerated vs never incarcerated | 2.86 (2.59 - 3.14) | 2013 | 2013 APH IBBA |
| OR of being HCV positive if ever incarcerated vs never incarcerated | 2.34 (2.14 - 2.56) | 2015 | 2015 APH IBBA |
| OR of being HCV positive if ever incarcerated vs never incarcerated | 2.58 (2.34 - 2.83) | 2017 | 2017 APH IBBA |
| OR of being HCV positive if female vs male | 1.01 (0.91 - 1.12) | 2013 | 2013 APH IBBA |
| OR of being HCV positive if female vs male | 0.92 (0.82 - 1.02) | 2015 | 2015 APH IBBA |
| OR of being HCV positive if female vs male | 0.88 (0.78 - 0.98) | 2017 | 2017 APH IBBA |
| OR of being HCV positive if <=25 vs >25 | 0.29 (0.26 - 0.33) | 2013 | 2013 APH IBBA |
| OR of being HCV positive if <=25 vs >25 | 0.24 (0.21 - 0.29) | 2015 | 2015 APH IBBA |
| OR of being HCV positive if <=25 vs >25 | 0.22 (0.18 - 0.27) | 2017 | 2017 APH IBBA |
| **Intervention Coverage** | | | |
| Proportion of HIV positive PWID on ART | 16.9% (13.0 - 19.0) | 2011 | 2011 APH IBBA |
|  | 41.0% (36.0 – 48.0) | 2013 | 2013 APH IBBA |
|  | 29.0% (26.6 - 33.4) | 2015 | 2015 APH IBBA |
|  | 40.7% (35.3 - 47.2) | 2017 | 2017 APH IBBA |
| OR of being on ART if in contact with NGO vs not in contact (among those HIV positive) | 3.03 (2.72 - 3.39) | 2014 | 2011/13/15/17 APH IBBAs |
| OR of being on ART if >25 vs <=25 (among those HIV positive) | 3.09 (1.95 - 4.90) | 2014 | 2011/1315/17 APH IBBAs |
| Proportion of PWID in contact with NGOs | 46.2% (44.4 - 47.1) | 2011 | 2011 APH IBBA |
|  | 49.3% (48.6 - 50.1) | 2013 | 2013 APH IBBA |
|  | 37.0% (36.1 – 38.0) | 2015 | 2015 APH IBBA |
|  | 38.8% (37.6 - 39.3) | 2017 | 2017 APH IBBA |
| Proportion of PWID in contact with NGOs who have had contact for less than 2 years | 60.2% (59.6 - 61.2) | 2011 | 2011 APH IBBA |
|  | 57.8% (55.5 - 60.5) | 2013 | 2013 APH IBBA |
|  | 57.3% (54.6 - 62.5) | 2015 | 2015 APH IBBA |
|  | 35.2% (33.9 - 37.1) | 2017 | 2017 APH IBBA |
| OR of being an NGO contact if HIV positive vs HIV negative | 2.11 (2.00 - 2.23) | 2013 | 2009/11/13/15/17 APH IBBAs |
| OR of being an NGO contact if <=25 vs >25 | 0.45 (0.42 - 0.48) | 2013 | 2009/11/13/15/17 APH IBBAs |
| Proportion of PWID on OAT | 3.9% (3.6 - 4.6) | 2015 | 2015 APH IBBA |
|  | 4.8% (4.4 - 5.3) | 2017 | 2017 APH IBBA |
| OR of being on OAT if in contact with NGO vs not in contact | 8.00 (6.75 - 9.47) | 2016 | 2015/17 APH IBBAs |

Additional Results


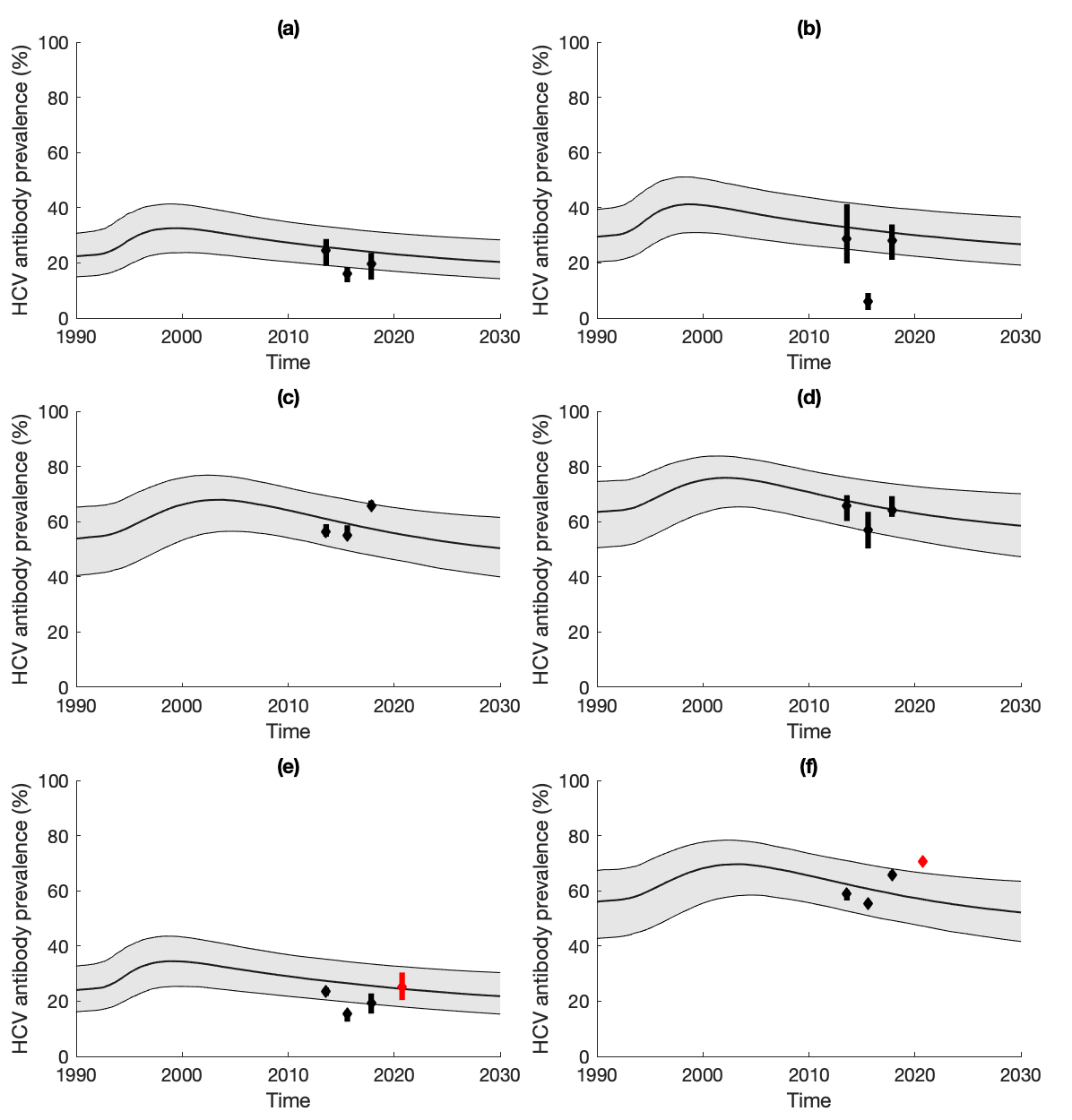


Supplementary Figure 1: *Status quo projections of community HCV antibody prevalence by age and gender. (a) young male PWID (<25); (b) young female PWID (<25); (c) older male PWID (>=25); (d) older female PWID (>=25); (e) younger PWID (<25); (f) older PWID (>=25) Black lines and grey shaded area show the median and 95%CrI of the baseline model fits. Data points with whiskers show data and their 95% CIs. Red data points show estimates from the 2020 IBBA survey which was not used for model parameterisation or calibration.*


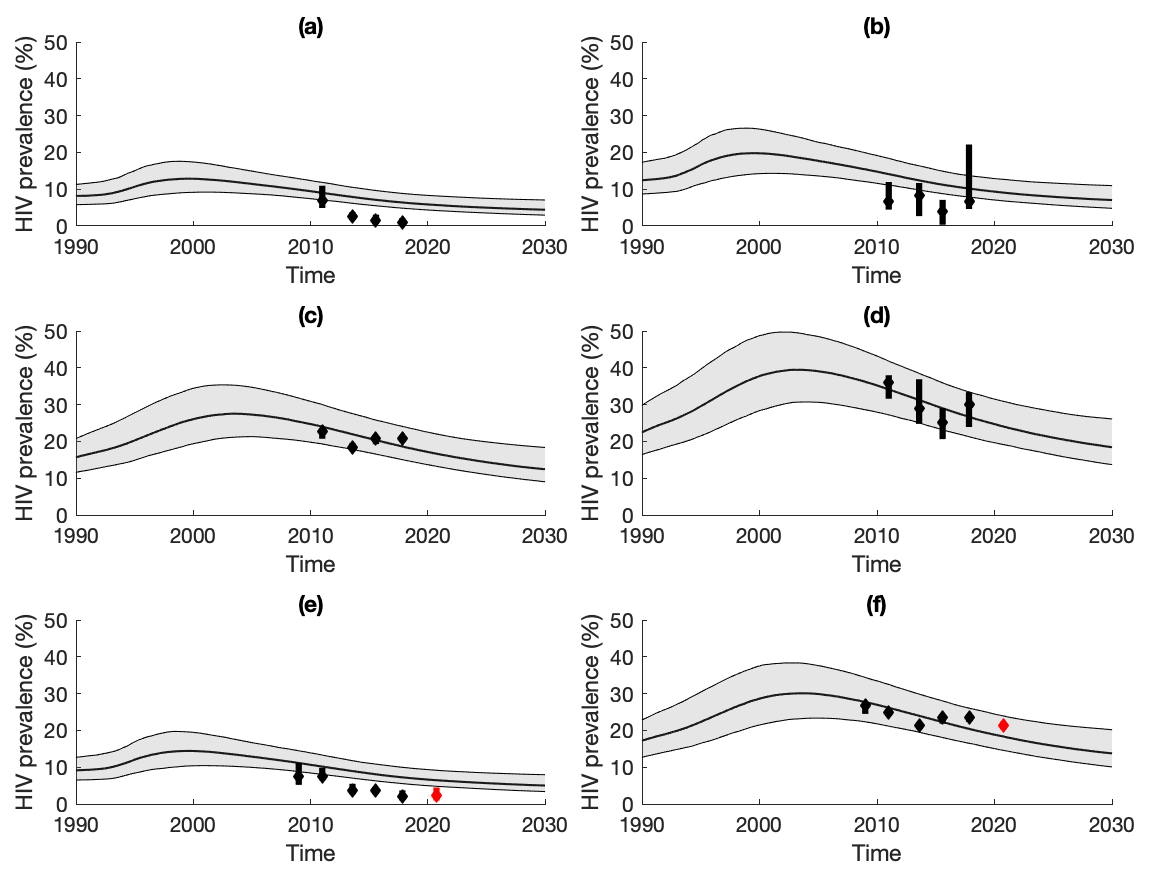


Supplementary Figure 2: *Status quo projections of community HIV prevalence by age and gender. (a) young male PWID (<25); (b) young female PWID (<25); (c) older male PWID (>=25); (d) older female PWID (>=25); (e) younger PWID (<25); (f) older PWID (>=25). Black lines and grey shaded area show the median and 95%CrI of the baseline model fits. Data points with whiskers show data and their 95% CIs. Red data points show estimates from the 2020 IBBA survey which was not used for model parameterisation or calibration.*


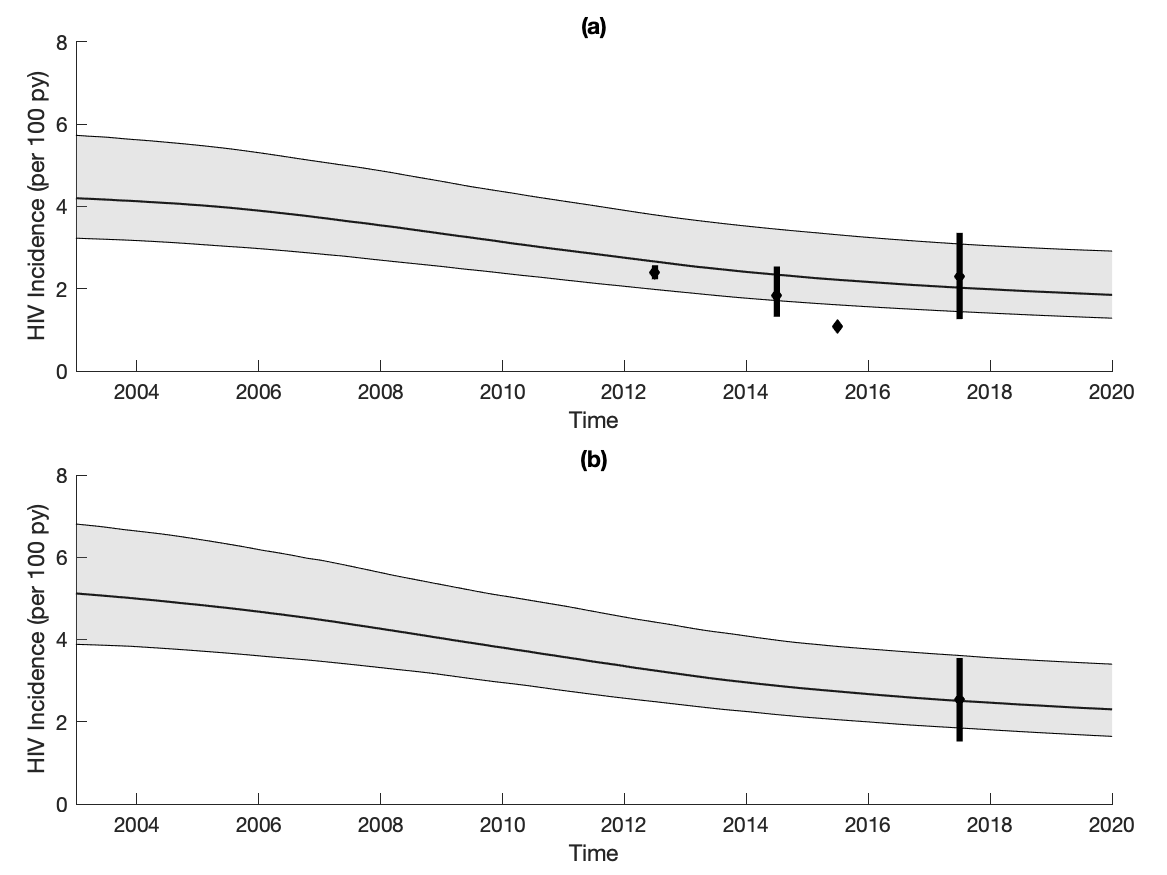


Supplementary Figure 3: *Status quo projections of HIV incidence among (a) contacts of NGOs and (b) Non-NGO contacts. Black lines and grey shaded area show the median and 95%CrI of the baseline model fits. Data points with whiskers show data and their 95% CIs – which were not used in model calibration but for model validation.*

**Supplementary Table 3:** Cost effectiveness of scaled-up levels of non-governmental organisation (NGO) provision over 2022-2026 compared to status quo. In both scenarios, NGO coverage continues at baseline levels after 2026. Table shows results using baseline cost assumptions and a discount rate of 3% per annum. Cells present median projections across 1,000 model fits along with 95% credibility intervals in parentheses. OAT = opioid agonist treatment. ART = antiretroviral therapy. DALY = disability-adjusted life year.

|  | **Status Quo** | **Scaled-up NGO 2022-2026** | **Incremental** |
| --- | --- | --- | --- |
| Cost of NGO  (Million $; 2022-2041) | 170  (132 - 214) | 241  (200-295) | 70  (53 - 99) |
| Cost of ART  (Million $; 2022-2041) | 145  (97 - 237) | 141  (95 - 233) | -3  (-7 - -0) |
| Cost of OAT  (Million $; 2022-2041) | 62  (41 - 95) | 84  (55 - 128) | 22  (13 - 38) |
| Total Costs  (Million $; 2022-2041) | 377  (303 - 503) | 467  (379 - 614) | 88  (64 - 131) |
| DALYs averted | - | - | 73,218  (46795 - 120,027) |
| Incremental cost-effectiveness ratio  (ICER; $ per DALY averted) | - | - | 1,204.2  (875.4 – 1602.0) |


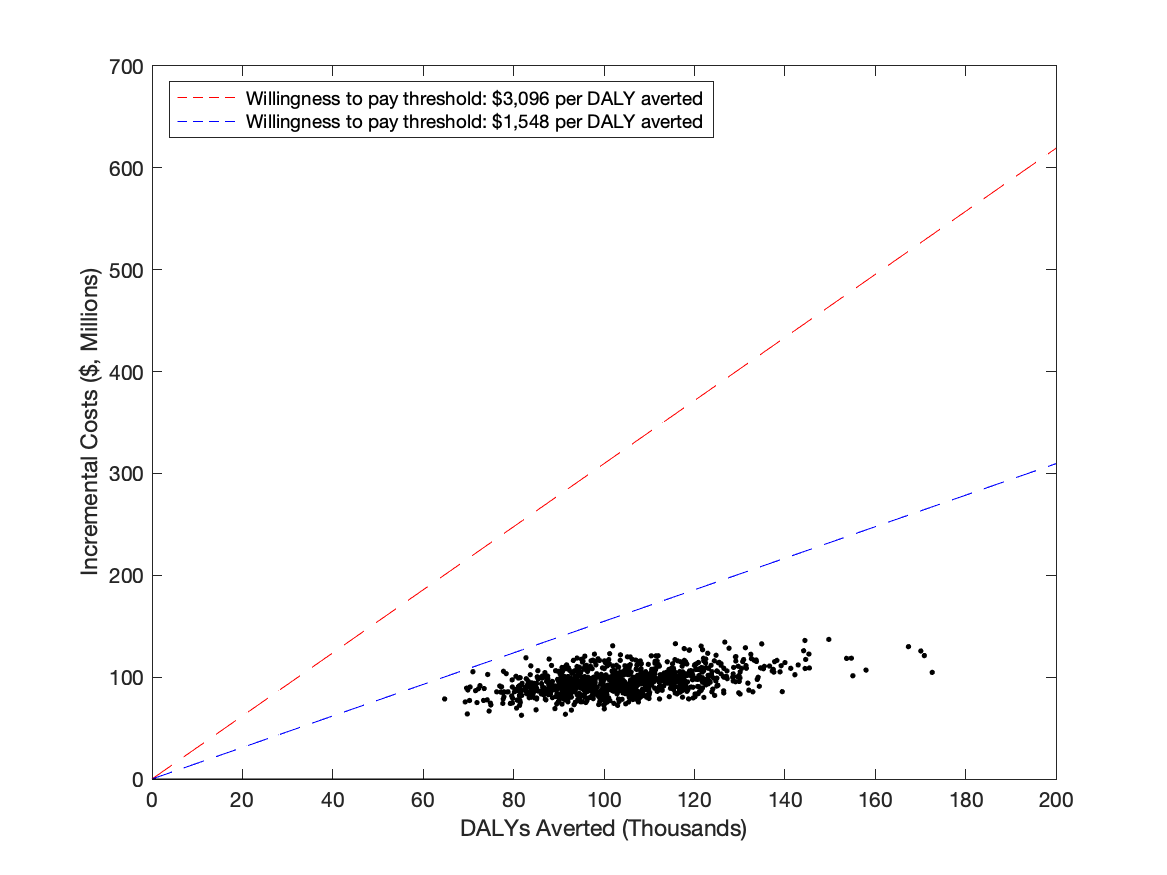


Supplementary Figure 4: *Cost-effectiveness plane for the Baseline cost-effectiveness analysis. Black points represent each model run. Dashed lines show the WTP threshold of 1xGDP (red) and 0.5xGDP (blue).*


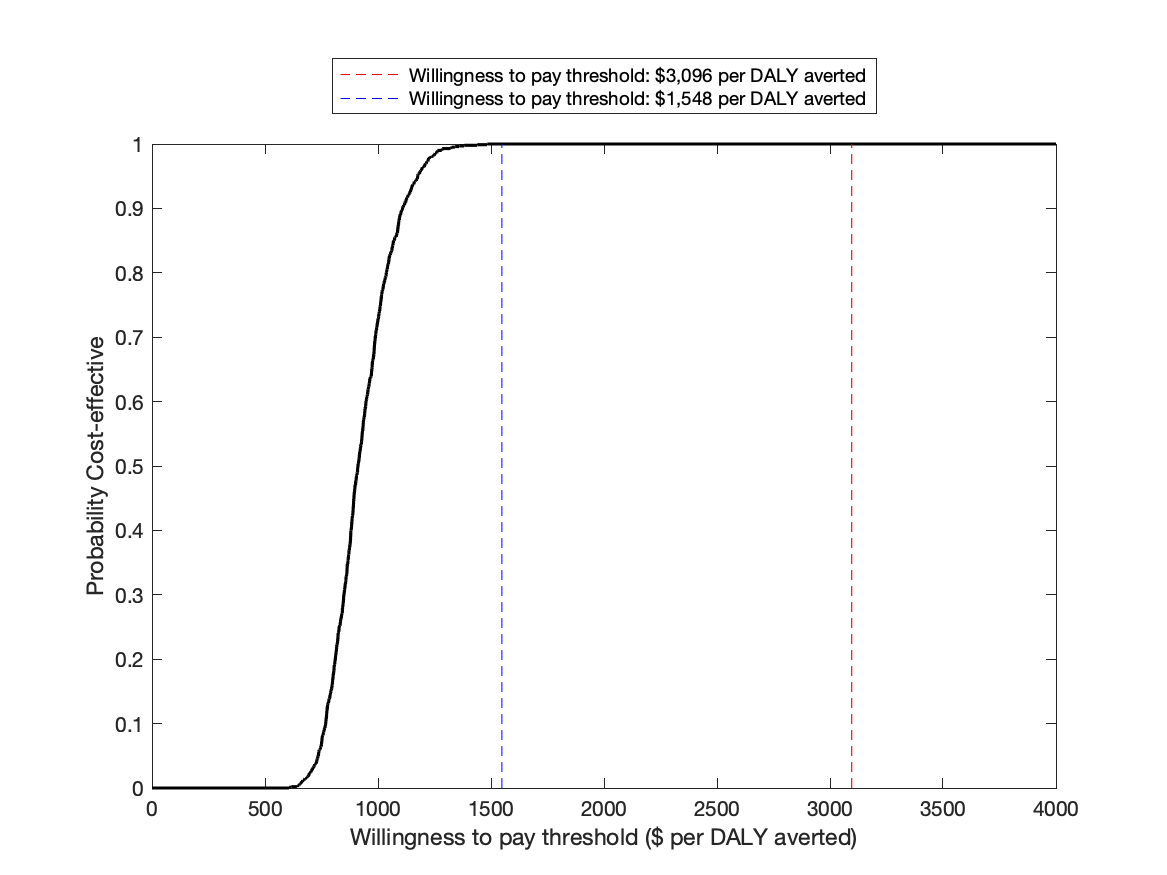


Supplementary Figure 5: *Cost-effectiveness acceptability curve for the Baseline cost-effectiveness analysis. Dashed lines show the WTP threshold of 1xGDP (red) and 0.5xGDP (blue).*

 *Supplementary Figure 6: Sensitivity analyses. Red bars show the median ICER in each of the sensitivity analyses. The solid black line shows the median baseline ICER. The dotted and dashed black lines show a willingness-to-pay threshold of 0.5 and 1xGDP (*US$1,548 *and US$3096), respectively.*

**References**

1. Quinn TC, Wawer MJ, Sewankambo N, Serwadda D, Li C, Wabwire-Mangen F, et al. Viral load and heterosexual transmission of human immunodeficiency virus type 1. Rakai Project Study Group. N Engl J Med. 2000;342(13):921-9.

2. Hughes JP, Baeten JM, Lingappa JR, Magaret AS, Wald A, de Bruyn G, et al. Determinants of per-coital-act HIV-1 infectivity among African HIV-1-serodiscordant couples. J Infect Dis. 2012;205(3):358-65.

3. International HIV/AIDS Alliance in Ukraine. Integrated Behavioral and Biological Assessment 2013. Available from: <https://aph.org.ua/wp-content/uploads/2016/07/zvit-IDU_obl_eng.pdf>.

4. International HIV/AIDS Alliance in Ukraine. Integrated Behavioral and Biological Assessment 2015. Available from: <https://aph.org.ua/wp-content/uploads/2015/05/Monitoryng-povedinky-SIN__Eng__Na-RED.pdf>.

5. International HIV/AIDS Alliance in Ukraine. Integrated Behavioral and Biological Assessment 2011. Available from: <https://aph.org.ua/wp-content/uploads/2016/08/idu_en_2011.pdf>.

6. International HIV/AIDS Alliance in Ukraine, Integrated Behavioral and Biological Assessment 2017.

7. Zelenev A, Shea P, Mazhnaya A, Meteliuk A, Pykalo I, Marcus R, et al. Estimating HIV and HCV prevalence among people who inject drugs in 5 Ukrainian cities using stratification-based respondent driven and random sampling. Int J Drug Policy. 2019;67:91-101.

8. Miller WC, Hoffman IF, Hanscom BS, Ha TV, Dumchev K, Djoerban Z, et al. A scalable, integrated intervention to engage people who inject drugs in HIV care and medication-assisted treatment (HPTN 074): a randomised, controlled phase 3 feasibility and efficacy study. Lancet. 2018;392(10149):747-59.

9. Hollingsworth TD, Anderson RM, Fraser C. HIV-1 transmission, by stage of infection. J Infect Dis. 2008;198(5):687-93.

10. Morgan D, Mahe C, Mayanja B, Okongo JM, Lubega R, Whitworth JA. HIV-1 infection in rural Africa: is there a difference in median time to AIDS and survival compared with that in industrialized countries? AIDS. 2002;16(4):597-603.

11. Public Health Center of Ukraine's Ministry of Health Statistics on the Number of Patients on ART in Ukraine, Informational Bulletin. Kyiv, Ukraine. 2016. Available from: <https://old.phc.org.ua/pages/diseases/hiv_aids/statistics/art>.

12. Murray M, Hogg RS, Lima VD, May MT, Moore DM, Abgrall S, et al. The effect of injecting drug use history on disease progression and death among HIV-positive individuals initiating combination antiretroviral therapy: collaborative cohort analysis. HIV Med. 2012;13(2):89-97.

13. May MT, Ingle SM, Costagliola D, Justice AC, de Wolf F, Cavassini M, et al. Cohort profile: Antiretroviral Therapy Cohort Collaboration (ART-CC). Int J Epidemiol. 2014;43(3):691-702.

14. Mukandavire C, Low A, Mburu G, Trickey A, May MT, Davies CF, et al. Impact of opioid substitution therapy on the HIV prevention benefit of antiretroviral therapy for people who inject drugs. AIDS. 2017;31(8):1181-90.

15. Nosyk B, Min JE, Evans E, Li L, Liu L, Lima VD, et al. The Effects of Opioid Substitution Treatment and Highly Active Antiretroviral Therapy on the Cause-Specific Risk of Mortality Among HIV-Positive People Who Inject Drugs. Clin Infect Dis. 2015;61(7):1157-65.

16. Grebely J, Raffa JD, Lai C, Krajden M, Conway B, Tyndall MW. Factors associated with spontaneous clearance of hepatitis C virus among illicit drug users. Can J Gastroenterol. 2007;21(7):447-51.

17. Fraser H, Mukandavire C, Martin NK, Hickman M, Cohen MS, Miller WC, et al. HIV treatment as prevention among people who inject drugs - a re-evaluation of the evidence. Int J Epidemiol. 2017;46(2):466-78.

18. Smith DJ, Combellick J, Jordan AE, Hagan H. Hepatitis C virus (HCV) disease progression in people who inject drugs (PWID): A systematic review and meta-analysis. Int J Drug Policy. 2015;26(10):911-21.

19. Thein HH, Yi Q, Dore GJ, Krahn MD. Natural history of hepatitis C virus infection in HIV-infected individuals and the impact of HIV in the era of highly active antiretroviral therapy: a meta-analysis. AIDS. 2008;22(15):1979-91.

20. Limketkai BN, Mehta SH, Sutcliffe CG, Higgins YM, Torbenson MS, Brinkley SC, et al. Relationship of liver disease stage and antiviral therapy with liver-related events and death in adults coinfected with HIV/HCV. JAMA. 2012;308(4):370-8.

21. Shepherd J, Jones J, Hartwell D, Davidson P, Price A, Waugh N. Interferon alpha (pegylated and non-pegylated) and ribavirin for the treatment of mild chronic hepatitis C: a systematic review and economic evaluation. Health Technol Assess. 2007;11(11):1-205, iii.

22. Lopez-Dieguez M, Montes ML, Pascual-Pareja JF, Quereda C, Von Wichmann MA, Berenguer J, et al. The natural history of liver cirrhosis in HIV-hepatitis C virus-coinfected patients. AIDS. 2011;25(7):899-904.

23. Merchante N, Giron-Gonzalez JA, Gonzalez-Serrano M, Torre-Cisneros J, Garcia-Garcia JA, Arizcorreta A, et al. Survival and prognostic factors of HIV-infected patients with HCV-related end-stage liver disease. AIDS. 2006;20(1):49-57.

24. Morgan RL, Baack B, Smith BD, Yartel A, Pitasi M, Falck-Ytter Y. Eradication of hepatitis C virus infection and the development of hepatocellular carcinoma: a meta-analysis of observational studies. Ann Intern Med. 2013;158(5 Pt 1):329-37.

25. Weller S, Davis K. Condom effectiveness in reducing heterosexual HIV transmission. Cochrane Database Syst Rev. 2001(3):CD003255.

26. Boily MC, Baggaley RF, Wang L, Masse B, White RG, Hayes RJ, et al. Heterosexual risk of HIV-1 infection per sexual act: systematic review and meta-analysis of observational studies. Lancet Infect Dis. 2009;9(2):118-29.

27. Dumchev K, Dvoryak S, Chernova O, Morozova O, Altice FL. Retention in medication-assisted treatment programs in Ukraine-Identifying factors contributing to a continuing HIV epidemic. Int J Drug Policy. 2017;48:44-53.

28. Low AJ, Mburu G, Welton NJ, May MT, Davies CF, French C, et al. Impact of Opioid Substitution Therapy on Antiretroviral Therapy Outcomes: A Systematic Review and Meta-Analysis. Clin Infect Dis. 2016;63(8):1094-104.

29. Platt L, Minozzi S, Reed J, Vickerman P, Hagan H, French C, et al. Needle syringe programmes and opioid substitution therapy for preventing hepatitis C transmission in people who inject drugs. Cochrane Database Syst Rev. 2017;9:CD012021.

30. MacArthur GJ, Minozzi S, Martin N, Vickerman P, Deren S, Bruneau J, et al. Opiate substitution treatment and HIV transmission in people who inject drugs: systematic review and meta-analysis. BMJ. 2012;345(oct03 3):e5945.

31. Larney S, Toson B, Burns L, Dolan K. Effect of prison-based opioid substitution treatment and post-release retention in treatment on risk of re-incarceration. Addiction. 2012;107(2):372-80.

32. Werb D, Kerr T, Marsh D, Li K, Montaner J, Wood E. Effect of methadone treatment on incarceration rates among injection drug users. Eur Addict Res. 2008;14(3):143-9.

33. Degenhardt L, Grebely J, Stone J, Hickman M, Vickerman P, Marshall BDL, et al. Global patterns of opioid use and dependence: harms to populations, interventions, and future action. Lancet. 2019;394(10208):1560-79.

34. Sordo L, Barrio G, Bravo MJ, Indave BI, Degenhardt L, Wiessing L, et al. Mortality risk during and after opioid substitution treatment: systematic review and meta-analysis of cohort studies. BMJ. 2017;357:j1550.

35. Toni T, Welch D, Strelkowa N, Ipsen A, Stumpf MP. Approximate Bayesian computation scheme for parameter inference and model selection in dynamical systems. J R Soc Interface. 2009;6(31):187-202.

36. Dumchev K., Varetska O., Berleva H. Estimating the number of people who inject drugs in Ukraine: a decade of experience. IHR Conference Abstract 423.

37. Estimation of the Size of Populations Most-at-Risk for HIV Infection in Ukraine in 2016”. Alliance for Public Health, Kyiv, Ukraine. Available at: <https://aph.org.ua/wp-content/uploads/2016/12/o4sn16.pdf>.

38. Alliance for Public Health. Estimation of the Size of Populations Most-at-Risk for HIV Infection in Ukraine in 2019. Available from: <https://aph.org.ua/wp-content/uploads/2019/06/Otsinka-chiselnosti_32200.pdf>.
